# Biofunctional Excipients: Their Emerging Role in Overcoming the Inherent Poor Biopharmaceutical Characteristics of Drugs

**DOI:** 10.3390/pharmaceutics17050598

**Published:** 2025-05-01

**Authors:** Milad Reda Qelliny, Wesam W. Mustafa, Adel Al Fatease, Ali H. Alamri, Raid Alany, Hamdy Abdelkader

**Affiliations:** 1 Department of Pharmaceutics, Faculty of Pharmacy, Minia University, Minia 61519, Egypt; mila_reda@mu.edu.eg; 2 Department of Pharmaceutics, Faculty of Pharmacy, Minia National University, Minia 61768, Egypt; 3 Department of Pharmaceutics, College of Pharmacy, Al-Mustafa University, Baghdad 996X+JXC, Iraq; wessam-pharm@almustafauniversity.edu.iq; 4 Department of Pharmaceutics, College of Pharmacy, King Khalid University, Abha 62529, Saudi Arabia; afatease@kku.edu.sa (A.A.F.); aamri@kku.edu.sa (A.H.A.); 5 School of Pharmacy, Kingston University London, Kingston Upon Thames KT1 2EE, UK; r.alany@kingston.ac.uk

**Keywords:** pharmaceutical excipients, biofunctional excipients, smart polymers, lipids, amino acids, cyclodextrins, surfactants, pH-modifiers, pharmacokinetic profile

## Abstract

**Background/Objectives:** With advancements in biomaterial sciences, biofunctional excipients have emerged to focus on solving issues with the drugs’ inherent biopharmaceutical characteristics such as poor solubility, permeability, in vivo dissolution, and effective targeting. These advanced excipients significantly impact drug solubility, dissolution rates, absorption rates, permeation rates, penetration ability, targeting ability, and pharmacokinetic profiles. **Methods:** A literature review of recently published articles was prepared. Data were collected using scientific search engines. This review provided a detailed discussion of various biofunctional excipients including smart polymers, targeted polymers, bioadhesive polymers, lipids, amino acids, cyclodextrins, and biosurfactants. Each category was discussed in detail concerning its biofunctional applications, the mechanisms underlying these biofunctions, and examples of their effects on drug performance. **Results:** The data obtained indicated that the rapid advances in the manufacturing of pharmaceutical excipients have resulted in the development of a diverse array of smart or intelligent excipients that play a crucial role in enhancing inherent poor biopharmaceutical characteristics. **Conclusions:** These advancements have also facilitated the development of various drug delivery systems, including immediate, controlled, sustained, and targeted drug release systems. Also, numerous nano-based delivery systems have emerged utilizing the newly produced excipients.

## 1. Introduction

Pharmacologically active compounds or drugs commonly denoted as active pharmaceutical ingredients (APIs) are typically not directly used or administered in isolation to patients but are instead incorporated into meticulously formulated dosage forms. These pharmaceutical formulations serve as a platform or a carrier to ensure consistent and precise dosing, efficacy, safety, stability, and high levels of patient acceptance and adherence [1]. Initially, dosage forms were rudimentarily produced and manufactured by blending and/or mixing pharmacologically inert substances, known as excipients, with the API to achieve the desired volume of a suitable dosing unit. However, advancements in pharmaceutical technology have facilitated the identification and manufacturing of excipients that serve specific functions beyond mere volumetric adjustment. These functions include aiding in the production process of dosage forms and optimizing drug delivery from innovative formulations. Indeed, the roles of excipients in dosage forms encompass the various aspects and functions of the final product. These include API stability, manufacturing process, dose uniformity, efficient drug delivery into systemic circulation post-administration, ameliorating side effects, and favorable sensory properties to ensure maximal patient compliance [2]. However, many newly developed drug candidates suffer from poor water solubility and low oral bioavailability. These issues often lead to suboptimal therapeutic outcomes, increased dosage requirements, and limited clinical success.

By definition, excipients could be defined as a material or combination of materials that provides adequate bulk and ensures uniform content distribution, functioning as a carrier to integrate the API into the formulation [3]. In traditional and conventional methods, excipients, as mere inert components within medicinal formulations, have undergone significant transformations, evolving from simplistic, chemically and pharmacologically passive, and cost-effective excipients to indispensable functional constituents or excipients such as smart polymers, ensuring and optimizing formulation performance [2]. It is broadly acknowledged and recognized that the effectiveness of medications is influenced not only by the strength of the API and production techniques but also by the pharmaceutical characteristics of the excipient used in the formulation such as the material quality. Excipients are incorporated into APIs to enhance their stability and preservation, tonicity, and to streamline drug delivery, thereby fostering the development of highly effective medications [3,4].

With a plethora of over 1000 pharmaceutical additives, ranging from small molecules to intricate natural, semi-synthetic, and synthetic polymeric compounds sourced from diverse origins, various dosage forms can be formulated. Excipients are meticulously selected to fulfill a multitude of roles including augmenting the formulation volume, safeguarding the API stability, refining the precision and dosage accuracy of APIs, bolstering bioavailability, and facilitating API administration by improving the palatability and optimizing the feasibility of large-scale manufacturing processes [3,5].

With recent advancements in both pharmaceutical and medical fields, a wide range of APIs with inherent biopharmaceutical limitations, such as low solubility and restricted permeability, have been found post-marketing. This may result in erratic drug absorption, inconsistent bioavailability, irritation, and unfavorable pharmacokinetic profiles, consequently leading to suboptimal therapeutic outcomes and potential adverse drug reactions. Increasing recognition has surfaced regarding the multifaceted in vivo effects exerted by substances previously regarded as biologically inert excipients. This awareness has led to the emergence of vast biofunctional excipients such as smart lipids, polymers, and other safe, natural additives capable of modulating drug solubility, permeability, release kinetics, and pharmacokinetic behaviors. Examples of such biofunctional excipients include in situ forming polymers, cyclodextrins, and amino acids, among others [2,6].

The current review undertakes a thorough investigation of biofunctional excipients, delving into their diverse classifications, roles, functionalities, limitations, biological impacts, and recent advancements within this new field. Through extensive exploration, this review aims to explore the significance of biofunctional excipients in pharmaceutical formulations, shedding light on their nuanced effects and potential implications for drug delivery and therapeutic outcomes.

## 2. Biofunctional Excipients

**Figure 1:**
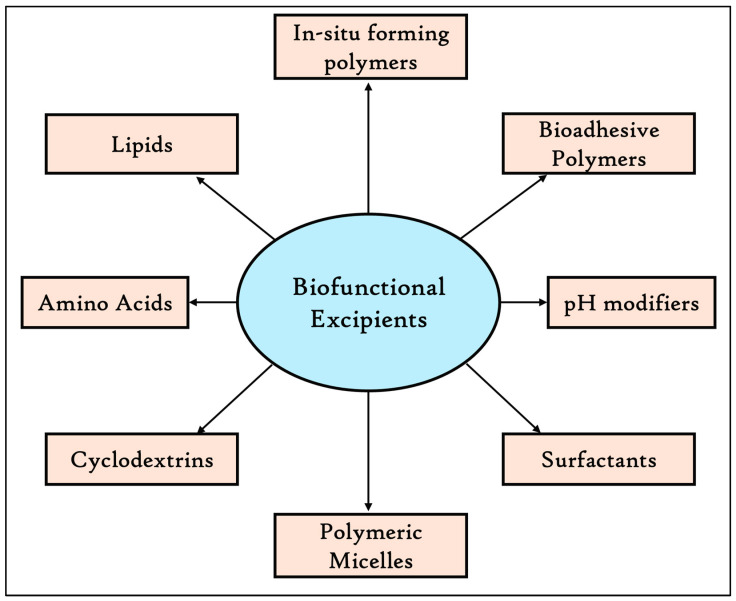
Different classes of biofunctional excipients investigated in the present review. The biofunctional excipients include a wide range of pharmaceutical materials such as polymers, lipids, surfactants, micelles, cyclodextrins, amino acids, pH-modifiers, and in situ forming polymers.

According to Hamman and Steenekamp (2012), the word “Functional” is used to indicate that a material has a specific property or function that could improve manufacturability [2]. The term biofunctional is not a standard term but could be defined, according to the available data, as excipients in pharmaceutical formulations with inherent biological activity or functionality beyond their conventional responsibilities [7]. Biofunctional excipients actively contribute to the therapeutic efficacy or safety of the drug, in contrast to conventional excipients, which are often employed to help in the manufacturing process, stabilize the API, or increase the drug’s distribution and absorption. By interacting biologically, they can improve the drug’s stability, bioavailability, release profiles, pharmacokinetic profiles, and overall efficacy. Biofunctional excipients, for example, can be agents that deliver a regulated or targeted release of the drug or chemicals that work as permeability enhancers to boost drug absorption or as antioxidants to prevent drug degradation. These excipients are essential for creating pharmaceutical formulations that are more patient-friendly and effective, especially for medications with low bioavailability or solubility. Biofunctional excipients include a wide range of materials such as smart and bioadhesive polymers, pH-modifiers, surfactants, polymeric micelles, cyclodextrins, amino acids, lipids, in situ forming polymers, and others (Figure 1) [7]. In conclusion, biofunctional excipients are substances that confer biological functions, significantly influencing drug performance parameters such as solubility, absorption, permeability, bioavailability, biodistribution, and pharmacokinetic profiles.

Biofunctional excipients, also known as biopharmaceutical excipients, are pivotal in the formulation of biologic therapeutics, significantly enhancing the bioavailability, solubility, and tonicity of APIs [8]. Beyond their role in therapeutic applications, these excipients function as preservatives, antioxidants, and bulking agents. Notably, biopharmaceutical excipients remain inert, refraining from any chemical interactions with the final product while ensuring its stability. However, biofunctional excipients may interact with biological components to enhance drug absorption, targeting, or therapeutic action. Key examples of such excipients include cellulose derivatives, polysaccharides, plant-based compounds, and synthetic substances. Inorganic minerals and copolymers, commonly utilized in biopharmaceuticals, contribute to the inertness and stability of drug formulations [8]. These excipients play a crucial role in improving drug solubility and permeability, particularly for APIs with poor water solubility. Additionally, they help to modulate the drug’s release profile, facilitating more consistent therapeutic outcomes. By optimizing drug absorption, biofunctional excipients enhance bioavailability, leading to more effective treatments. Furthermore, they bolster the chemical, physical, and microbial stability of drugs, mitigating risks of degradation and preserving product efficacy [7]. It is important to know that biofunctional excipients are facing critical challenges such as regulatory aspects, and interactions with biological components, such as metabolic enzymes.

## 3. Biofunctional Excipients Added to Enhance Drug Solubility and Dissolution

**Figure 2:**
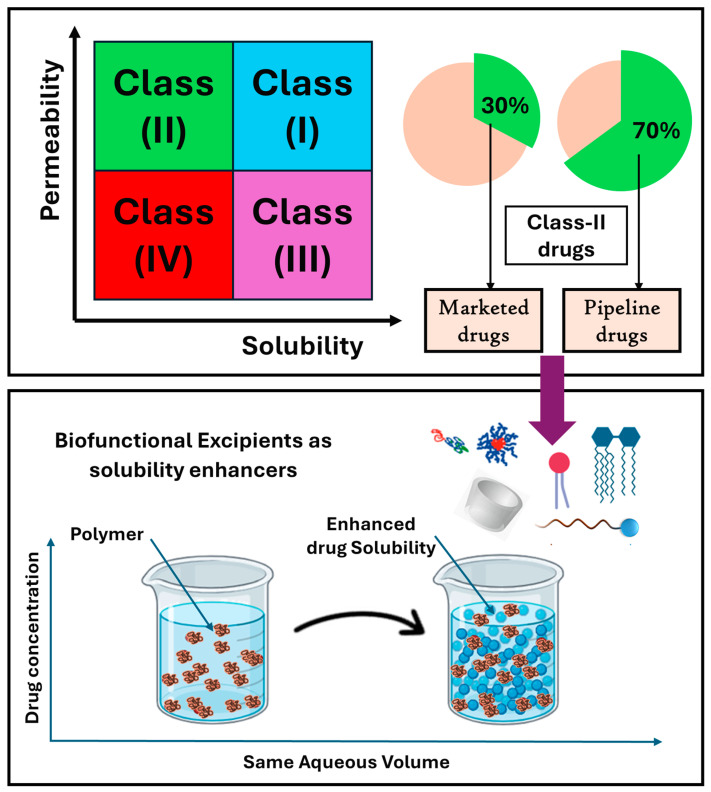
Biopharmaceutical classification of drugs (BCS) and role of polymers as solubility enhancers. According to BCS, most commonly available drugs in market (30%) and drugs in pipeline pathway (70%) are Class II drugs (higher permeability, low solubility drugs) [11]. For this reason, polymers could be used as solubility enhancers via different techniques such as conjugation, complexation, solid dispersion techniques, and others.

Drug solubility is a critical parameter in pharmaceutical formulations and particularly in drug delivery via the oral route. The oral bioavailability of APIs largely depends on their absorption through the gastrointestinal (GI) tract [9]. The Biopharmaceutics Classification System (BCS) offers a framework that links drug absorption to solubility and intestinal permeability. Marketed drugs are fairly distributed across the BCS classifications; Class I compounds (high permeability and high solubility) are the most common [10]. However, newer drug candidates show a shift in trend. Only 5–10% fall into Class I, while the majority (60–70%) are categorized as Class II (high permeability and low solubility). This trend continues in the development pipeline, where up to 90% of pipeline drugs belong to Class II and Class IV (low solubility and low permeability), which results in poor and erratic solubility and inconsistent clinical performance [11,12] (Figure 2). Poor solubility in these classes is often linked to properties such as high crystallinity or high lipophilicity.

The reason for this unexpected disparity in modern drug discovery lies in the influence of genomics and molecular biology, which have prompted the adoption of combinatorial chemistry, automated high-throughput screening (HTS), and computational drug design methodologies. From the 1990s to the 2000s, pharmaceutical companies significantly increased their capacity to generate molecular targets and compound data points expanded dramatically from 200,000 to over 50 million. Many promising HTS leads for disease treatment exhibit high drug potency, primarily driven by hydrophobic interactions in drug–receptor binding or enzyme-binding sites [13]. Consequently, these pipeline drugs often possess high hydrophobicity (or lipophilicity), which limits their water solubility and their suitability for oral delivery (Figure 2) [11].

For the preparation of oral drug delivery systems, the drug solubility should be balanced with drug permeability. The limited drug solubility and slow dissolution rates of poorly water-soluble drugs in the aqueous GIT environment frequently result in inadequate bioavailability. The rate of drug absorption may be dissolution-rate-limited in cases where the dissolution rate is slower than the permeation rate. On the other hand, solubility-rate-limited absorption is capped by its solubility [14]. Compounds categorized under BCS Class II and Class IV exhibit low solubilities and slow, inadequate, and inconsistent dissolution, presenting significant challenges for systemic drug delivery. These challenges encompass incomplete drug release from the dosage form, limited bioavailability, and substantial interpatient variability [15]. Rather than absorption, the drug release from the dosage form and its solubility in gastric juices are the rate-limiting steps in the case of BCS Class II and Class IV medications. Therefore, increasing solubility directly correlates with enhanced bioavailability for these drugs [16,17]. Traditionally, multiple techniques have been performed to enhance drug solubility such as salt formation (diclofenac sodium, Amlodipine besylate, and Atorvastatin calcium), complexation mechanisms (piroxicam–cyclodextrin), particle size reduction (ibuprofen and griseofulvin), nanoemulsions (Propofol, cyclosporine, and testosterone), and lyophilization (Vancomycin and Meropenem) [18]. The previously mentioned technologies for enhancing drug solubility require precise concentrations of crude materials and other contributing factors. In contrast, techniques for enhancing solubility by reducing surface tension using detergents and surfactants are more cost-effective and require less specialized expertise. However, their use is limited by potential adverse reactions such as hemolytic effects at higher concentrations, as seen with agents like sodium lauryl sulfate (SLS) and saponins, which pose risks to in vivo safety [19,20].

### 3.1. Functional Polymers

Recently, several polymers have been identified as biofunctional excipients due to their dual roles in pharmaceutical manufacturing and enhancing biofunctionality, such as improving drug solubility. These biofunctional excipients aid in the manufacturing of pharmaceutical products while also providing critical functions like solubility enhancement, which is essential for enhancing the bioavailability of poorly soluble drugs (Table 1). Available polymers include polyvinyl alcohol (PVA), polyvinyl pyrrolidone (PVP), polyvinylpyrrolidone−vinyl acetate copolymers (PVP-VA), hydroxypropyl cellulose (HPC), hydroxypropyl methylcellulose (HPMC), hydroxypropyl methylcellulose acetate succinate (HPMCAS), carboxymethyl cellulose (CMC), polyethylene glycol (PEG), and methacrylate copolymers such as Eudragit polymers, and specifically Eudragit E (Table 1) [11,21].

Polyvinyl alcohol (PVA, sometimes known as PVOH) is a synthetic polymer known for its water solubility, which varies with its degree of hydrolysis and polymerization [22]. Partially hydrolyzed PVA (85–89%) exhibits higher aqueous solubility and is more commonly employed in pharmaceutical drug delivery systems. In contrast, fully hydrolyzed PVA (98–99%) demonstrates lower solubility and is predominantly used in film formation and coating applications. PVA is a copolymer composed of vinyl acetate and vinyl alcohol segments, produced and synthesized via vinyl acetate polymerization and subsequent partial hydrolysis. The degree of hydrolysis and polymerization determines PVA’s solubility and physical properties [23]. These factors influence the balance between its hydrophilic (hydroxyl) and hydrophobic (acetyl) groups, affecting its solubility properties [24]. Due to this unique combination of groups, PVA can form micelle-like structures, creating optimal conditions for solubilizing poorly soluble drugs. Furthermore, as a non-ionic, pH-independent polymer, PVA maintains consistent drug solubilization performance throughout the GIT, leading to the reduced variability and enhanced bioavailability of the drugs [25]. PVA enhances drug solubility via forming micelle-like structures and could be used with multiple drugs such as Amenamevir, nifedipine, tacrolimus, and itraconazole [25,26] via different manufacturing techniques such as amorphous solid dispersions (ASDs) [25,26], hot-melt extrusions [27], and 3D printing technologies [28,29].

**Figure 3:**
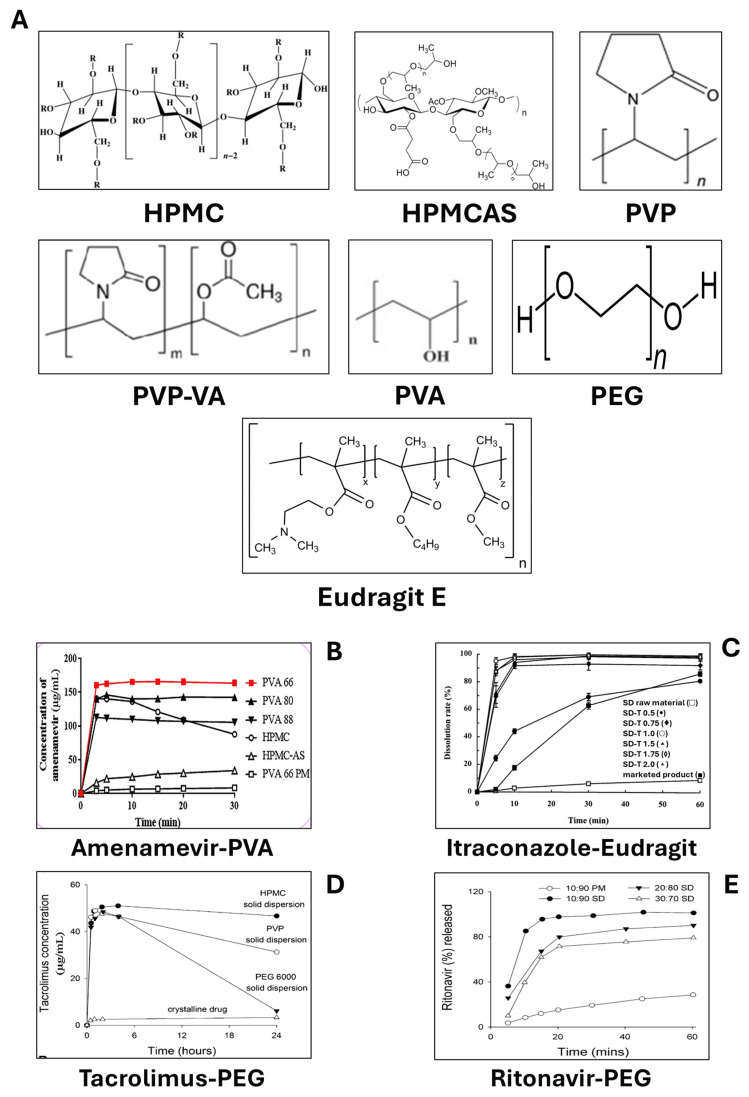
Structure of the most used polymers as solubility enhancers. (**A**) Polymers include cellulose derivatives such as hydroxypropyl methylcellulose (HPMC), hydroxypropyl methylcellulose succinate (HPMCAS), polyvinyl pyrrolidone (PVP), polyvinyl pyrrolidone–vinyl acetate (PVA-VA), polyvinyl alcohol (PVA), polyethylene glycol (PEG), and methacrylate copolymers such as Eudragit E. (**B**) Dissolution rates of Amenamevir with PVA and HPMC solid dispersions. (**C**) Dissolution rates of itraconazole–Eudragit E solid dispersions. (**D**) Tacrolimus solid dispersion with PEG, HPMC, and PVP. (**E**) Cumulative drug release from ritonavir solid dispersion with PEG. This photo was adapted from the following sources [25,38,61].

Polyvinyl pyrrolidone (PVP), also known as povidone, is a neutral homopolymer [11]. PVP is supplied under various trade names such as Kollidon and Plasdone (Figure 3). These excipients are available in different molecular weights, which are designated by kinematic viscosity values (referred to as K-values according to USP Convention nomenclature) in aqueous solutions. Generally, higher kinematic viscosity, or K-values, indicate higher molecular weights and higher glass transition temperatures (Tg) [30]. These characteristics are crucial for ensuring the processability and stability of amorphous solid dispersion (ASD) products. PVP has been utilized either independently or in combination with PEG to improve the solubility of poorly aqueous-soluble drugs, particularly those classified under BCS Class-II, such as nabilone (enhanced pharmacological activity) [11], troglitazone [11], and celecoxib (inhibition of crystal growth) [31,32] (Table 1).

In the same way, the modified PVP copolymers such as polyvinyl pyrrolidone–vinyl acetate (PVP-VA) can be employed due to their increased polymer hydrophobicity and reduced Tg, typically ranging between 103 and 106 °C [33]. The incorporation of vinyl acetate (VA) segments in the polymer backbone structure enhances hydrophobicity and imparts a moderate plasticizing effect, facilitating thermal processing. Although both PVP and PVP-VA possess a group of carbonyl oxygens that function as intermolecular hydrogen bond acceptors, PVP-VA proves to be a more efficient crystal growth inhibitor for poorly soluble drugs such as celecoxib and efavirenz [31]. PVP-VA may exhibit a faster dissolution rate and dissolve more rapidly in water compared to the drug component in ASDs, which can potentially lead to rapid drug recrystallization under non-sink, supersaturation conditions [34]. PVP-VA has been used in marketed products such as HIV anti-viral drugs: lopinavir and ritonavir [32] (Table 1).

Polyethylene glycols (PEGs), also referred to as macrogols, are non-toxic, inert, biodegradable, and hydrophilic polyethers composed of repeated ethylene glycol units [-(CH_2_CH_2_O)n] (Figure 3) [19]. The high polarity of PEG augments its hydrophilicity, facilitating hydrophobic interactions with hydrophobic drugs, which is essential for solubilization. Consequently, PEG exhibits substantial solubility in various organic and inorganic solvents [35]. Hydrophobic drugs struggle to disrupt the strong lattice structure of water; therefore, co-solvents like PEG enhance solubilization by reducing the polarity of the solvent system. PEG can influence the crystallization of APIs, potentially delaying, promoting, or having no effect on crystallization [36]. For instance, in a PEG matrix, the induction time of ibuprofen crystallization was reduced compared to that of the pure drug. This was attributed to crystalline PEG serving as a heterogeneous nucleation site, thereby expediting the crystallization of ibuprofen [37]. PEG polymers have been utilized to improve the solubility of various drugs, including griseofulvin, fenofibrate, and nimodipine [11,38,39,40,41,42] (Table 1).

Ionic copolymer excipients, notably provided by Evonik under the brand name Eudragit^®^ and represented by methacrylate-based polymers, play a critical role in various ASDs. These polymers are synthesized and manufactured via (meth) acrylic acid copolymerization with amino alkyl methacrylates, methacrylic esters, or ammonioalkyl methacrylates, offering a diverse range of pH-independent, pH-dependent, and time-dependent polymers. There are a wide range of Eudragit polymers available, such as Eudragit S100, L100, E100, RS100, and RL 100 [11,43]. The Eudragit L 100 and S 100 variants, which differ in their degree of ionizable carboxylic acid content, control drug delivery based on the intestinal pH, with L 100 dissolving at a lower pH (5.5) compared to S 100 (7.0). Research by Maghsoodi and Sadeghpoor demonstrated the pH-dependent dissolution behavior of Eudragit S 100 in solid dispersion microparticles containing the NSAID piroxicam [43], indicating that the dissolution rate of piroxicam is significantly increased and enhanced (Table 1). The Eudragit E series, a methacrylate/aminoester copolymer which is soluble and dissolvable in acidic pHs, such as in gastric fluid up to pH 5.0, enhances gastric delivery, as evidenced by spray-dried ASDs of itraconazole with Eudragit E 100, which significantly increased drug solubility in simulated gastric media compared to other hydrophilic polymers (Figure 3). Overall, the diverse range of Eudragit polymers, including anionic, cationic, and neutral methacrylate copolymers, allows for the optimization of ASD’s physical and chemical properties, enhancing drug release performance and bioavailability [44,45] (Table 1).

Cellulose, a naturally occurring polysaccharide, consists of β(1→4)-linked D-glucose (anhydroglucose) monomers (Figure 3) [46]. Typically, cellulose derived from plants has a high molecular weight, often exceeding 1 × 10^6^ Daltons [47]. Due to its molecular structure featuring three hydroxyl groups per glucose unit, cellulose is highly functionalizable, enabling the creation of various derivatives with specific properties tailored for pharmaceutical applications [48,49,50,51]. Several cellulose derivatives are widely used in drug formulation, including methylcellulose (MC), cellulose acetate phthalate (CAP), HPMC, and HPMCAS. Among these, HPMC and HPMCAS are particularly significant for their roles in enhancing drug solubility and stability in ASDs [11,32]. The amphiphilic properties of these polymers and specifically for HPMCAS allow poorly soluble drugs to interact with the hydrophobic regions of the polymer and for the hydrophilic regions to stabilize the colloidal aqueous solution [52]. In the same way, a cellulose derivative polymer “HPMC” is synthesized by cellulose reacting with propylene oxide, methyl chloride, and caustic soda. HPMC is classified into four grades based on the weight percentage of methoxy and hydroxypropyl groups: HPMC 1828, HPMC 2208, HPMC 2906, and HPMC 2910. The grades differ in viscosity, affecting their dissolution rates and processing properties. HPMC 2910, renowned for its solubility in a broad spectrum of solvents, is especially effective in creating supersaturated drug solutions and delaying the nucleation of crystalline APIs [53,54,55].

HPMCAS is manufactured via HPMC esterification with acetic and succinic anhydrides (Figure 3). The three grades of HPMCAS—716, 912, and 126—are differentiated by their acetate and succinate content. HPMCAS exhibits amphiphilic properties, broad organic solvent solubility, and a relatively low Tg of about 120 °C when compared to HPMC, which enhances its processability during hot-melt extrusion and solid dispersion preparation. These characteristics make it a versatile polymer for enhancing the solubility of hydrophobic drugs [11]. Studies by Curatolo et al. and Friesen et al. have shown that HPMCAS-based ASDs can substantially enhance the solubility and subsequently the oral bioavailability of poorly aqueous-soluble drugs by creating stable dispersions and inhibiting drug crystallization. The high Tg of HPMC (155−180 °C) contributes to its effectiveness in providing physical stability to ASDs but may limit its processability at industrial scales. In contrast, HPMCAS, with its lower Tg and strong drug–polymer interactions, is highly effective in maintaining drug supersaturation and enhancing bioavailability, despite potential challenges in manufacturing due to the variability in the chain extension of hydroxypropyl groups [49,52]. HPMC and other derivatives are used for the formulation of multiple poorly soluble drugs such as verapamil, nifedipine, itraconazole, nilvadipine, tacrolimus, everolimus, ivacaftor, posaconazole, ziprasidone, vemurafenib, and telaprevir [11,38,39,40,41,42] (Table 1).

**Table 1:** Examples of biofunctional polymers for enhanced drug solubility.

Drug	Uses	BCS Class	Polymer	Formulation ^1^	Biofunctional Applications	Ref
Verapamil	Anti-hypertensive	BCS II	HPMC	HME	Enhanced in vivo drug solubilization and drug bioavailability via formation of SD and gel formation upon water contact.	[38]
Itraconazole	Anti-fungal	BCS II		SD		[42]
Nilvadipine	Anti-hypertensive	BCS II		NA		[42]
Tacrolimus	Immunosuppressant	BCS II		SD		[56]
Nabilone	Anti-emetic	BCS II	PVP	HME	Enhanced in vivo drug solubilization and stabilization via inhibition of poorly soluble drug crystal growth.	[42]
Troglitazone	Anti-diabetic	BCS II		HME		[42]
Celecoxib	Analgesic	BCS II		NA		[57]
Griseofulvin	Anti-fungal	BCS II	PEG	HME	Enhanced in vivo drug solubilization via the higher hydrophilicity that allow interactions with hydrophobic drugs.	[58]
Fenofibrate	Anti-hyperlipidemia	BCS II		SD		[42]
Nimodipine	Anti-hypertensive	BCS II		SD		[42]
Ivacaftor	Cystic fibrosis	BCS II	HPMCAS	SD	Enhanced in vivo drug solubilization and stabilization via the colloidal nature of the polymer when ionized and via the hydrophobic substitutes.	[59]
Posaconazole	Anti-fungal	BCS II		HME		[42]
Ziprasidone	Anti-psychotic	BCS II		Mixing		[32]
Vemurafenib	Anti-cancer	BCS II		Co-precip.		[60]
Telaprevir	Anti-viral	BCS II		SD		[42]
Ritonavir	Anti-viral	BCS IV	PVP-VA	HME	Enhanced in vivo drug solubilization, stabilization, and bioavailability via the formation of micelle-like structures, creating optimal conditions for solubilizing poorly soluble drugs.	[42]
Lopinavir	Anti-viral	BCS IV		HME		[42]
Amenamevir	Anti-viral	BCS IV	PVA	Ball Milling		[25]
Itraconazole	Anti-fungal	BCS II	Eudragit E	SD	Enhanced drug dissolution	[32,61]
Ibuprofen	Analgesic	BCS II	PEG-PVP	Evaporation		[62]
Piroxicam	Analgesic	BCS II	Eudragit S	MP		[43]

^1^ HME: hot-melt extrusions; SD: spray drying; Sol./Stab.: enhanced solubility and stability, Co-precip: co-precipitation; MP: microparticles; and NA: not available. Most of the previously mentioned drugs are already marketed as tablets.

### 3.2. Functional Lipids and Lipid Derivatives

Lipids are fundamental in the oral delivery of various APIs due to their structural versatility and role as natural dietary components. They not only provide energy but also serve as carriers for poorly aqueous-soluble vitamins and nutrients, interacting favorably with bile components [12]. Upon digestion by lipases, lipids break down into monoglycerides and fatty acids, which interact with lipophilic drugs, enhancing their solubility and absorption via different mechanisms such as emulsion formation, mixed micelle formation, and the promotion of lymphatic transport. Lipid-based excipients, derived from vegetable oils, fatty acids, or waxes, include triacylglycerols, phospholipids, and lipophilic vitamins [63,64].

**Figure 4:**
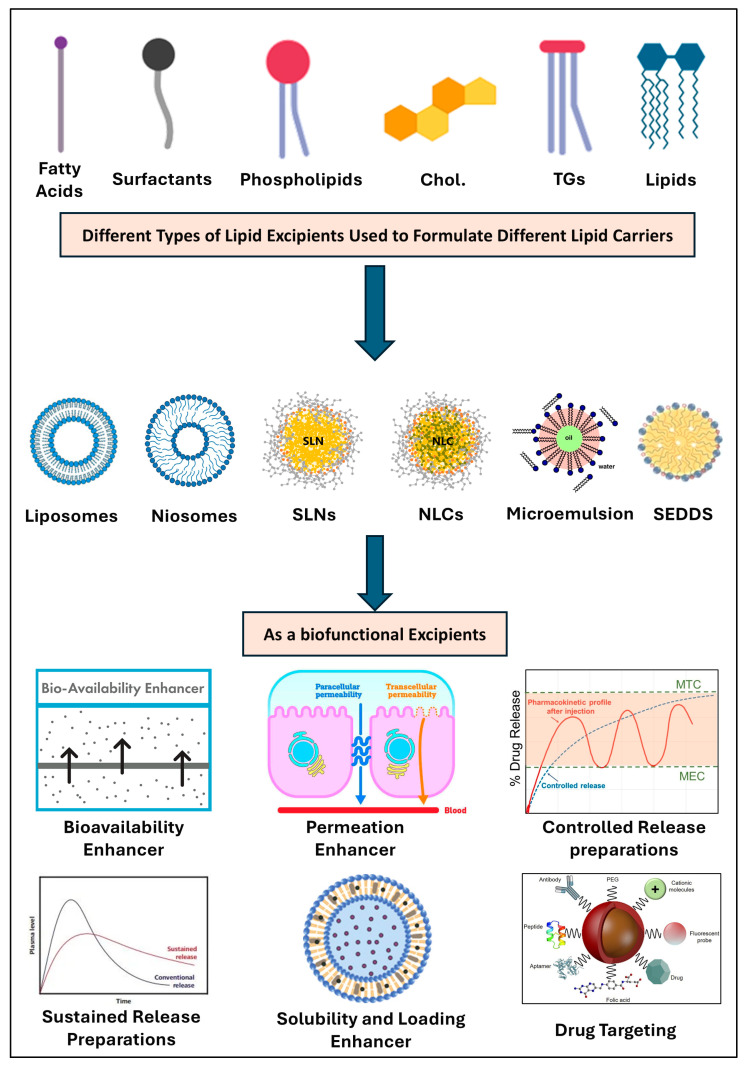
Different types of lipid excipients utilized for the formulation of a wide range of lipid nanocarriers. Lipid excipients are integral to the formulation of various lipid nanocarriers, encompassing fatty acids, triglycerides (TGs), surfactants, emulsifiers, co-emulsifiers, phospholipids, cholesterol, and mixed lipids. These excipients are employed in a diverse array of lipid-based nanoformulations, including liposomes, solid lipid nanoparticles (SLNs), nanostructured lipid carriers (NLCs), micro- and nanoemulsions, and self-emulsifying drug delivery systems (SEDDSs). Additionally, lipid derivatives find applications in other formulations such as oily solutions, suspensions, nanocapsules, hot-melt preparations, and solid dispersions. The extensive range of available lipid excipients and nanoformulations offers numerous biofunctional advantages, such as enhanced bioavailability, solubility, permeation, and drug loading capacity. These advancements contribute to improved drug release profiles, the development of both controlled and sustained drug delivery systems, and the formulation of targeted carriers that enhance therapeutic efficacy while minimizing side effects.

To enhance the bioavailability of poorly soluble medications, lipid-based excipients are frequently utilized in a variety of oral dosage forms, including oily solutions, oily suspensions, emulsions, microemulsions, solid lipid nanoparticles (SLNs), liposomes, nanostructured lipid carriers (NLC), and self-emulsifying drug delivery systems (SEDDSs) (Figure 4) [12,65]. This system’s ability to create intraluminal colloidal phases, which maintain the drug’s solubility, is what makes it effective. This enhancement is primarily due to the pre-dissolution of the drug in the formulation, which obviates the necessity for this GIT process. By creating localized supersaturation near the intestinal epithelium through the uptake of fatty metabolites, they also expedite the rate of medication absorption [66]. Also, suitable lipid vehicles could minimize or prevent drug crystallization in the GIT [32]. On the other hand, lipids, along with certain amphiphilic polymers, can act as decoys by interacting with proteins that mediate these processes, thereby facilitating the drug’s penetration into enterocytes and circumventing this barrier to bioavailability [67,68]. Once absorbed, most molecules, including drugs, exit the cellular environment primarily through the portal blood supply, driven by the high blood flow that effectively facilitates this process [63]. Ultimately, lipid excipients improve drug partitioning within the epidermal lipids of the skin, thereby facilitating and expediting the penetration of drugs through and into human skin [69].

Lipid excipients are primarily classified into various classes such as triglycerides (TGs), mixed glycerides, water-soluble and insoluble surfactants, polar oils, co-solvents, and various other additives. Common co-solvents used alongside lipid excipients include ethanol, glycerol, propylene glycol, and PEG-400 for the preparation of lipid vesicles. Surfactants with an intermediate hydrophilic–lipophilic balance (HLB) of 8–12, which are water-insoluble and adsorb at oil–water interfaces, are commercially available. Lipid-based synthetic surfactants, particularly non-ionic surfactants such as PEG-40 and PEG-60 hydrogenated castor oil, Polyoxyl 35 hydrogenated castor oil, Polysorbate 80 (Tween 80^®^), Polysorbate 20 (Tween 20^®^), glyceryl monooleate, Labrafil M1944, caprylocaproyl macrogol-8 glycerides (Labrasol^®^), and Linoleoyl Polyoxyl-6 glycerides (Labrafil^®^ M2125CS), are extensively utilized as lipid excipients. Additionally, water-insoluble surfactants like propylene glycol monolaurate (Lauroglycol™ FCC), propylene glycol monocaprylate (Capryol^TM^ 90), and Polyglyceryl-3 Dioleate, as well as water-soluble surfactants such as Polyoxyl Stearate and Polyoxyethylene Lauryl Ether, are widely incorporated into lipid-based formulations [70].

Multiple studies have extensively investigated the biofunctional role of lipid excipients and their effects on drug solubility, absorption, permeability, and bioavailability. For example, Kim et al. conducted a study comparing indomethacin oral bioavailability following the administration of an indomethacin-loaded SEDDS composed of 70% ethyl oleate and 30% Tween 85 versus an indomethacin suspension. The results demonstrated that the SEDDS formulation led to a significant 57% increase in the area under the curve (AUC 0−12 h) compared to the indomethacin suspension when administered orally to rats [71]. Menzel et al. (2018) reported the development of an exenatide-encapsulated SEDDS that exhibited higher mucous permeation capabilities, achieving an oral bioavailability of 14.6% in comparison to subcutaneous injection [72].

Microemulsion systems have proven highly beneficial across various pharmaceutical applications due to their significant enhancements in drug therapeutic activity and safety while reducing cytotoxic effects [73,74,75]. For instance, Allermyl^®^ utilizes a microemulsion system with a mix of oils, monosaccharides, and piroctone olamine, which facilitates high permeation through the epidermal cells by mimicking cellular membranes [76,77]. Solvium^®^ employs a microemulsion for ibuprofen, combining PEG-8 caprylic/capric glycerides and ethanol to improve oil loading and drug solubilization, resulting in a stable and translucent formulation [78,79]. Sandimmune Neoral^®^ uses anhydrous ethanol and lipophilic solubility enhancers to create a self-microemulsifying system for cyclosporine A, enhancing drug solubilization and preventing precipitation [78,79]. Norvir^®^ and Fortovase^®^ are microemulsions for HIV treatment, with Norvir^®^ utilizing PEG-35 castor oil and ethanol to stabilize the drug in an oil phase, while Fortovase^®^ employs a mix of medium-chain mono- and diglycerides and DL-alpha tocopherol to form a microemulsion upon contact with body fluids. Recent studies showed that both drugs exhibit minimal clinical activity over hard gel capsules [80,81]. These examples illustrate the versatile role of microemulsions in improving drug delivery and efficacy (Table 2).

**Table 2:** Application of different lipid biofunctional excipients in drug delivery systems.

Drug	Lipid Excipient ^1^	BCS Class	Formulation	Biofunctional Application	Route	Ref.
Cyclosporine	Mixed lipids	BCS IV	Soft capsules	Improved drug solubilization	Oral	[78]
Ritonavir	Mint oil, PEG–castor	BCS IV	Microemulsion	In vivo drug solubilization	Oral	[82]
Cinnarizine	Oleic acid	BCS II	Solution	Enhanced oral bioavailability (4-F)	Oral	[83]
Vitamin D3	LCT, MCT, and SCT	BCS IV	Solution	More BV in LCT>MCT>SCT	Oral	[84]
Danazol	Glycerol MO	BCS II	Emulsion	4-fold increase in bioavailability	Oral	[85]
Acyclovir	Labrafac, labrasol	BCS III	Microemulsion	13-fold increase in BV compared to tablets	Oral	[86]
Simvastatin	Capryol 90	BCS II	SEDDS	1.5-fold increase in BV	Oral	[87]
Carvedilol	Labrafil M1944CS	BCS II	SEDDS	4-fold increase in BV	Oral	[88]
Exenatide	Cre., Labrafil, Cap.	BCS III	SEDDS	14% enhancement in BV	Oral	[72]
Indomethacin	Ethyl oleate	BCS II	SEDDS	57% increase in AUC	Oral	[71]
Ibuprofen	PEG-8 Capryic TGs	BCS I	Microemulsion	Enhanced stability and BV	Oral	[79]
Ritonavir	PEG-35 castor oil	BCS II	Microemulsion	Minimal enhancement in BV	Oral	[81]
Saquinavir	MCT	BCS II	Microemulsion	Minimal enhancement in BV	Oral	[46]
Fenofibrate	Mixed lipids	BCS II	Hard capsules	In vivo drug solubilization	Oral	[70]

^1^ PEG: polyethylene glycol, LCT: long-chain triglyceride, MCT: medium-chain triglyceride, SCT: small-chain triglyceride, MO: mono-oleate, BV: oral bioavailability, F: fold, SEDDS: self-emulsifying drug delivery system, Cre.: Cremophor EL, and Cap.: Capmul-PG8.

### 3.3. Functional Amino Acids (AAs)

Recently, amino acids (AAs) have garnered significant and considerable attention in drug delivery and pharmaceutical excipient research owing to their advantageous properties. Throughout the past decade, the capacity of AAs to enhance drug properties has been firmly recognized [89]. Innovative approaches in drug delivery system development have increasingly focused on combining applicable molecules. The structural characteristics of amino acids (AAs), such as the α-carboxylate group, α-amino group, and diverse side chains, render them highly suitable for establishing bonds with drug molecules. This allows AAs to participate in various intermolecular interactions, such as hydrogen bonding, hydrophobicity, and ionic interactions [90]. As a result, AAs are regarded as effective low-molecular-weight excipients capable of binding to biological drug receptors [91]. Consequently, AAs are promising co-formers in drug formulations, providing a novel platform for enhancing drug properties like solubility, dissolution, permeation, stability, and therapeutic efficacy [92,93,94].

Amino acids (AAs) enhance drug solubility and absorption through multiple mechanisms. Basic amino acids such as arginine and lysine form salts with acidic drugs, improving solubility. Neutral amino acids like proline and glycine act as hydrotropes, enhancing drug solubility through non-ionic interactions. Certain amino acids also modulate the local pH to favor drug dissolution. Additionally, some amino acids facilitate absorption by interacting with specific transporters, including L-type amino acid transporters 1 (LAT1) and 2 (LAT2) [95,96].

The low water solubility of certain drugs (BCS II/IV) presents significant challenges for the pharmaceutical industry, affecting oral absorption, drug permeation, formulation, pharmacokinetic profiles, and bioavailability [97]. To overcome these challenges, the co-formulation of drugs with highly water-soluble compounds, such as AAs, has emerged as a promising strategy. Numerous studies have demonstrated that AAs can act as hydrophilic carriers, improving the aqueous solubility and dissolution rates of drugs [98,99]. For instance, the combination of indomethacin with amino acids like L-arginine (ARG) or L-lysine (LYS) using freeze-drying techniques has been shown to enhance drug solubility and dissolution rates. Arginine and lysine increased indomethacin solubility by 10,000- and 2296-fold, respectively [100]. AAs have also been employed as salt-forming agents to modify drug properties, as seen in the creation of highly soluble salts such as ibuprofen arginate, which improve the pharmacokinetic profiles of drugs [101].

Also, AA complexes with quercetin, rutin, and bendazac enhance the solubility to factors ranging from 68- to 433-fold [102]. Additionally, AAs have demonstrated superior relative bioavailability compared to traditional carriers like cyclodextrins, particularly in enhancing drug absorption through mechanisms beyond just solubility enhancement [103]. Furthermore, ternary systems incorporating AAs have shown potential in significantly increasing the solubility of poorly soluble drugs [104] (Table 3). Recent research has investigated the application of AAs as ion pairs to enhance the solubility and permeability of small molecules, providing a biodegradable, low-toxicity, non-irritating, and stable alternative for drug formulation. Additionally, the capacity of AAs to transport across cells may promote the facilitated transport of drug–amino acid complexes using AA carriers (following ion pair formation), thereby enhancing permeation. Several studies have examined the application of AAs as ion pairs to increase the solubility and permeability of small molecules [105,106,107]. However, the utilization of AAs as ion pairs for enhancing the solubility and permeability of proteins and macromolecules remains largely underreported [108].

### 3.4. Functional Cyclodextrins (CDs)

**Figure 5:**
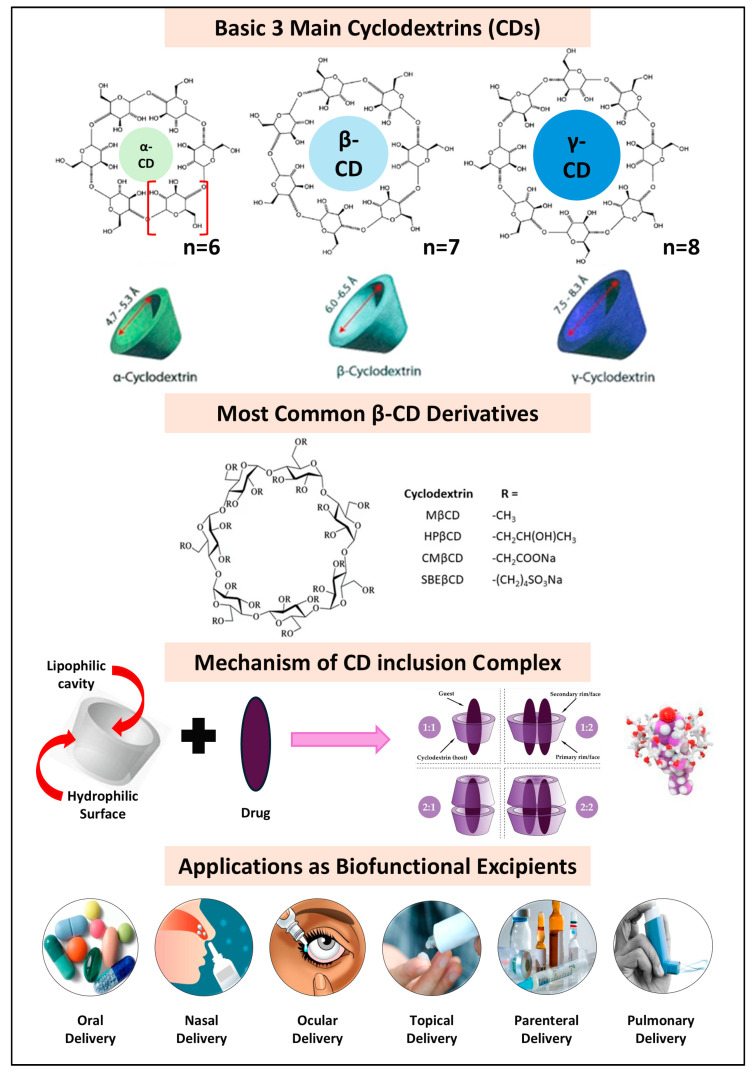
Chemical structure of basic cyclodextrins (α-, β-, γ-CDs) with the average lipophilic cavity size. The chemical structure of the most common β-CD derivatives, the mechanism of CD–drug inclusion complexes, and finally the wide range of CD pharmaceutical applications. **HP-β-CD**, hydroxypropyl-β-cyclodextrin; **M-β-CD**, Methyl-β-cyclodextrin; **CM-β-CD**, carboxymethyl-β-cyclodextrin; **SBE-β-CD**, sulfobutylether-β-cyclodextrin.

Cyclodextrins (CDs) serve as one of the most common biofunctional pharmaceutical excipients capable of improving the aqueous solubility of various drugs by forming water-soluble drug–CD complexes. These compounds are biocompatible and biodegradable cyclic oligosaccharides composed of six, seven, or eight (α-1,4)-linked D-glucopyranoside units, corresponding to α-, β-, and γ-CD, respectively. The three primary CDs (α-, β-, and γ-CDs), commonly referred to as “parent CDs,” along with their complexes, tend to exhibit limited water solubility, particularly in the case of β-CD. In response to this challenge, various chemically modified CD derivatives with improved water solubility have been synthesized and manufactured [124,125] (Figure 5). Moreover, the modification of CDs includes multiple simple techniques such as esterification [126], oxidation [127], substitution reactions [128], deoxy CDs [128], and O-substituted CDs [129]. These modifications increase the aqueous solubility of CDs by disrupting intramolecular hydrogen bonds and reducing crystallinity, resulting in amorphous forms that are more soluble in water.

Cyclodextrins (CDs) offer a diverse array of biofunctional properties such as enhanced drug solubility, drug dissolution properties, permeability, drug stability, mucoadhesion properties, and drug targeting. In oral drug delivery systems, CD inclusion complexes vary significantly depending on the administration route and specific physicochemical challenges [130]. Currently, CDs and their chemical derivatives are predominantly utilized for oral drug administration and delivery systems due to their capacity to enhance bioavailability by prolonging dissolution time and increasing release rates [131]. Naturally derived α-, β-, and γ-CDs, with their large central cavities, facilitate the formation of inclusion complexes with hydrophobic pharmaceutical compounds, improving the ease of oral administration (Table 4). Various CDs, including α-, β-, γ-cyclodextrin, and their derivatives like sulfated-α/β CD and 2-HP-β-CD, are employed in oral hygiene products to neutralize malodorous volatile sulfur compounds and improve drug palatability by reducing gastrointestinal irritation and bitterness “taste masking” [132]. Additionally, CDs can form “host–guest” complexes, interacting with gatekeeper proteins on taste buds to mask bitterness [133]. Recent studies have emphasized CDs as valuable excipients for enhancing water solubility, dissolution rates, and stability in various oral drug formulations [134,135] (Table 4).

CDs are highly effective in developing parenteral formulations, particularly for drugs with low aqueous solubility, as the majority of parenteral products are aqueous solutions [136]. The formation of water-soluble drug–CD inclusion complexes enhances drug solubilization, stability, and bioavailability by prolonging circulation time [137]. Among CDs, HP-β-CD and SBE-β-CD are preferred for parenteral use due to their superior water solubility, lower systemic toxicity, and good human tolerance [138]. Compared to co-solvent and solvent approaches, which may cause drug precipitation upon dilution and are therefore less suitable for parenteral products [139], CD-based systems offer significant advantages. CDs can self-assemble into NPs that improve encapsulation efficiency and drug loading, addressing common NP limitations [140]. These CD-based NPs often incorporate polymers to enhance stability, forming nanogels or micelle-like structures through various methods [141]. CDs also play a critical role in constructing smart and multifunctional delivery systems by interacting with numerous molecules that have small hydrophobic-like amino acids and peptides, thereby stabilizing proteins and enhancing their solubility and permeability [142]. In gene therapy, CDs serve as carriers for nucleic acids or genes, improving membrane permeation and reducing immunogenicity [143,144]. Additionally, CDs are used to develop functional nanomaterials through polymer conjugation, although comprehensive toxicological and biocompatibility evaluations are necessary for clinical applications [145]. From the previously discussed data, CDs have great promise in biofunctional uses and vast pharmaceutical applications compared to other excipients and could be applied to different administration routes (Table 4).

**Table 4:** List of different biofunctional applications of cyclodextrin–drug complexes.

Drug	CD Type	BCS Class	Formulation	Route	Biofunctional Applications	Ref.
Repaglinide	HP-β-CD	BCS II	Complex	Oral	Enhanced oral bioavailability	[146]
Cilostazol	β-CD	BCS II	Complex		Enhanced oral bioavailability	[147]
Albendazole		BCS IV	Solid matrix		Enhanced dissolution rate	[148]
Piroxicam	HP-β-CD	BCS II	Gel		Enhanced release and permeation	[149]
Disulfiram	HP-β-CD	BCS IV	Solution	Ocular	Enhanced ocular bioavailability	[150]
Tacrolimus		BCS II	Solution		Enhanced in vivo drug solubility	[151]
Curcumin		BCS IV	Solution		Improved solubility and release	[152]
Nepafenac		BCS II	Suspension		Increased trans-corneal permeation	[153]
Alprostadil	α-CD	BCS IV	Powder	Injection	Enhanced drug aqueous solubility and enhanced in vivo bioavailability	[136]
Letermovir	HP-β-CD	BCS II	Concentrate			
Amiodarone	SBE-β-CD	BCS II	Solution			
Aripiprazole		BCS II	Solution			

### 3.5. Functional Surfactants

Surfactants are organic molecules distinguished by their dual chemical structure, comprising a polar head group that interacts with polar phases and a nonpolar tail group that associates with nonpolar phases [154,155,156]. This distinctive structural composition enables surfactants to effectively lower the surface and interfacial tension between multiple phases [156]. Moreover, the self-assembly capability of surfactants in solution can result in the formation of micelles, which may vary in size from nanometers to micrometers. Their amphiphilic nature renders surfactants highly versatile and applicable in a broad spectrum of pharmaceutical products [156].

**Figure 6:**
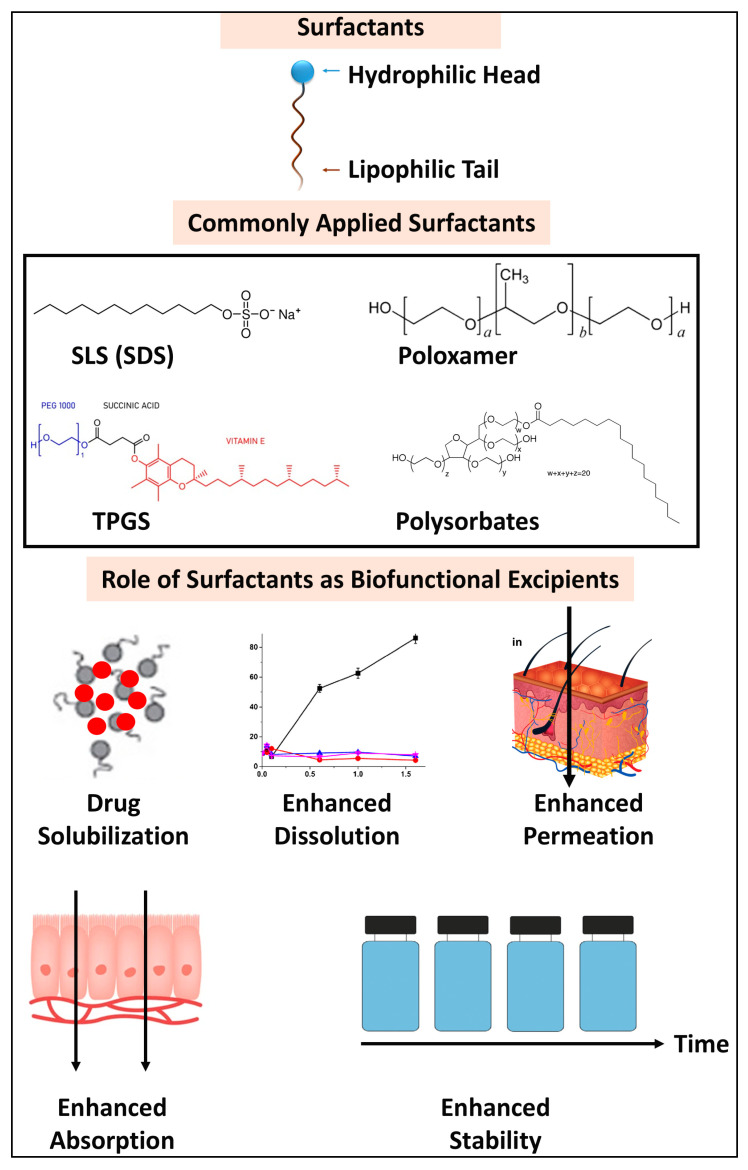
Application of surfactants as biofunctional excipients. The most used surfactants are sodium lauryl sulfate (SLS), poloxamers, d-α tocopherol polyethylene glycol succinate, and Polysorbates. Surfactants have a great impact on drug solubilization, dissolution rates, permeation, absorption, skin penetration, and drug stability.

From the perspective of drug solubility, the incorporation of surfactants can lower surface tension while simultaneously enhancing the solubility of drugs in either organic or aqueous solvents [157]. When dissolved in a liquid, surfactant molecules are drawn to the surface, where their presence can alter surface tension [157]. At low concentrations, surfactants adsorbed at the surface significantly modify the system’s free energy [158]. Once the surfactant molecules fully occupy the interface or surface area, they begin to aggregate, forming micelles (Figure 6). Micelles are nanoscale structures consisting of surfactant molecules, where the hydrophobic segments make up the core and the hydrophilic segments form the outer shell, typically ranging from 20 to 80 nm in diameter [159]. This micellar structure effectively dissolves drugs with low aqueous solubility. Therefore, surfactants can act as great drug solubilizers alone or in a combination with other excipients in solid dispersions or SEDDSs (Table 5).

Moreover, surfactants have a crucial role in improving the solubility and dissolution rates of pharmaceuticals that are poorly soluble in water (Figure 6), especially in solid dispersion systems. To facilitate more effective wettability, surfactants are added to solid oral dosage forms to lower the interface energy barrier between the dissolving media and the API. Surfactants are amphiphilic compounds with molecular structures that include both hydrophilic (polar) and hydrophobic (non-polar) functional groups. This amphiphilic nature allows poorly soluble drugs to interact with the hydrophobic portions of surfactants, enhancing their solubilities, while the hydrophilic parts stabilize the micellar structure in aqueous solutions [160,161]. Also, it has been demonstrated that much better medication release occurs when they are incorporated throughout the manufacturing process instead of being used as an extragranular excipient. A microenvironment rich in surfactants is created around the medicine during dissolution when surfactants like poloxamer and SLS (Figure 6) are thoroughly combined with solid dispersions [162]. This increases the aqueous solubility of the drug. This contrasts with the negligible impact that surfactants added extragranularly show. The capacity of surfactants to stop drug recrystallization, build hydrophobic barriers around drug particles, and speed up dissolving by causing a supersaturated state in aqueous media are is thought to be responsible for the improved solubility, dissolution rate, and oral bioavailability [163].

Multiple studies have explored the great impact of surfactants on drug absorption and bioavailability. For example, poloxamer 407 and Cremophor RH40-containing polymeric carriers were used by Lang et al. to create SDs of the poorly water-soluble medication itraconazole, which they found to release and supersaturate more quickly in aqueous environments. From in vitro dissolution data, approximately 20 mg, 17.5 mg, and 5 mg of itraconazole were released after 2 h with poloxamer 407, Cremophor RH40, and HPMCAS alone, respectively [164]. Pouton et al. explored how a formulation prepared with surfactants has been demonstrated to form a hydrophobic barrier around the drug particles and form agglomerates, which may impede the recrystallization of the drug [165]. According to a related work by Liu Chen et al., SLS interacted with PVP-VA to produce a PVP-VA/SLS complex with a reduced critical aggregation concentration (CAC), which considerably raised sorafenib’s apparent solubility despite its poor water solubility. The combination of PVPVA and SLS increased the equilibrium solubility of sorafenib by approximately 50-fold and enhanced the initial dissolution rate of the sorafenib tablet; however, the presence of SLS notably decreased PVPVA’s capacity to sustain sorafenib supersaturation [166]. The impact of poloxamer 188 on the poorly water-soluble medication carbamazepine–soluplus SD which was made by the solvent casting process was investigated by Medarevic et al. [167]. According to their data, only 43% of pure carbamazepine dissolved within 60 min, whereas over 90% of carbamazepine was released from all SDs containing poloxamer during the same period.

The surfactant improved the pace of dissolving the formulation, but it also caused the amorphous carbamazepine to crystallize out during storage, which negatively affected the drug’s physical stability. A combination of PEG–Polysorbate 80 has been described by Morris et al. [168], Serajuddin et al. [169], and Law et al. [170] as a surface-active carrier for poorly soluble medications. It has enhanced the dissolving profile and solubility of medications that are not very soluble in water. According to a previous study, adding a concentration of 5% *w*/*v* of SLS considerably accelerated the rate at which celecoxib dissolved, with three times as much dissolution occurring in the first 20 min of the test. It has been demonstrated that SLS can make medications, including tramadol hydrochloride, gabapentin, diazepam, alprazolam, buspirone, methocarbamol, and acetaminophen, more soluble [160]. Only after the CMC was attained did the active component become more soluble [171]. In a study by Varma and Panchagnula [172], it was shown that adding d-α tocopherol polyethylene glycol succinate (TPGS—Figure 6) to a formulation containing the anti-cancer medication paclitaxel greatly improved the drug’s solubility and bioavailability. Once the CMC (0.2 mg/mL) of TPGS was attained, the aqueous solubility of paclitaxel rose significantly, and when coupled with 5 mg/mL TPGS, it increased 38-fold. There was a noticeable increase in drug absorption in rats given the paclitaxel–TPGS combination orally (Table 5) [172].

**Table 5:** List of different applications for surfactants as biofunctional excipients.

Drug	Surfactant ^1^	BCS Class	Formulation ^1^	Route	Biofunctional Applications	Ref.
Posaconazole	SLS	BCS II	SD	Oral	Enhanced drug bioavailability	[173]
Ezetimibe	Tween 80	BCS II	SD	Oral	Little effect on bioavailability	[174]
Ritonavir	Tween 80	BCS IV	SD	Oral	Enhanced drug bioavailability	[175]
Sorafenib	SLS	BCS IV	SD	Oral	Enhanced bioavailability	[176]
Ibuprofen	Poloxamer	BCS II	SD	Oral	Enhanced drug bioavailability	[177]
Docetaxel	Poloxamer	BCS IV	SD	Oral	Enhanced drug bioavailability	[178]
Ibuprofen	Poloxamer	BCS II	SD	In vitro	Enhanced dissolution rate	[179]
Desloratadine	Poloxamer	BCS II	SD	In vitro	Enhanced dissolution rate	[180]
Celecoxib	SLS	BCS II	Tablets	In vitro	Enhanced dissolution rate	[160]
Dutasteride	TPGS	BCS II	SD	Oral	Enhanced bioavailability	[181]
Buspirone	SLS	BCS II	Tablets	In vitro	Enhanced dissolution rate	[160]

**^1^** SLS, sodium lauryl sulfate; TPGS, d-α tocopherol polyethylene glycol succinate; SD; solid dispersion.

## 4. Biofunctional Excipients Added to Enhance Drug Penetration and Permeability

According to BCS, both Class III and Class IV drugs suffer from inadequate permeability. These compounds demonstrate exceptionally low oral bioavailability and are predisposed to significant inter- and intrasubject variability. Consequently, unless administered at minimal dosages, they are typically unsuitable candidates for oral drug delivery. However, according to certain estimates, approximately 5% of the most commercially successful orally administered pharmaceuticals worldwide fall within this classification (Class IV). Enhancing the bioavailability of BCS Class IV drugs by modifying their structures during lead optimization is impractical due to high costs, time constraints, and low success rates in drug discovery. Given the extensive resources required, formulation strategies play a crucial role in ensuring the effective oral delivery of these compounds. Furthermore, formulation strategies for enhancing Class III and IV permeability could be achieved via using biofunctional excipients or nanoformulations.

### 4.1. Bioadhesive Polymers

In the context of drug delivery, bioadhesion is characterized by the capability of a drug carrier system, often composed of polymeric materials, to adhere to biological tissues for an extended period. This adherence enhances the concentration gradient of the drug at the absorption site, thereby improving the drug absorption, drug uptake, drug penetration, and the bioavailability of the systemically delivered drug. The internal body membranes are lined with a thick, viscous, and gel-like substance called mucin, which is secreted by goblet cells and other specialized exocrine glands containing mucous cell acini, collectively forming the mucous membrane [182].

**Figure 7:**
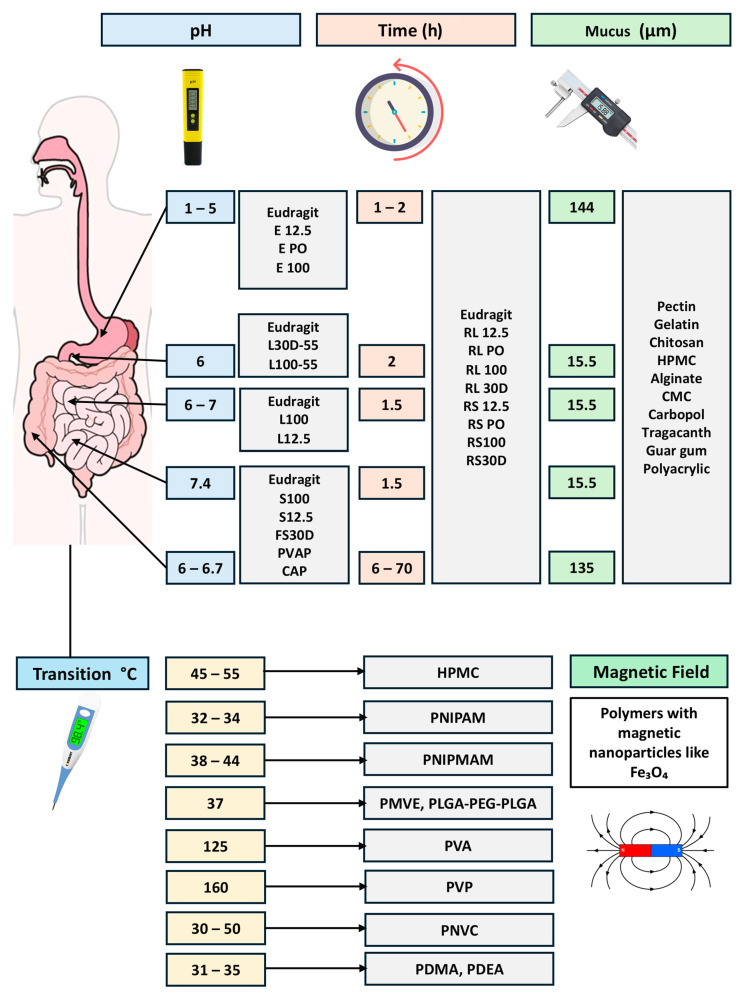
Different types of biofunctional polymeric excipients. The list of polymers includes pH-dependent polymers that are used for controlled and targeted drug delivery across different GIT compartments; time-dependent polymers that are used mainly for sustained release dosage forms; bioadhesive or mucoadhesive polymers that mainly enhance drug absorption across the GIT; and thermo-sensitive polymers with different transition temperatures that are widely applicable for various drug delivery systems. HPMC: hydroxypropyl methyl cellulose, CMC: carboxymethyl cellulose, PVAP: polyvinyl acetate phthalate, CAP: cellulose acetate phthalate, PNIPAM: poly (N-isopropylacrylamide), PNIPMAM: poly (N-isopropylmethylacrylamide), PMVE: poly (vinylmethylether), PLGA-PEG-PLGA: triblock poly lactoglycolic acid and polyethylene glycol, PVA: polyvinyl alcohol, PVP: polyvinyl pyrrolidine, PNVC: poly (N-vinylcaprolactam), PDMA: Poly(N,N-dimethylacrylamide), and PDEA: Poly(N,N-Diethyl Acrylamide).

These mucous membrane-covered areas represent potential sites (gastrointestinal tract, genital system, and ocular system) for the attachment of bioadhesive drug delivery systems which employ specific bioadhesive carriers, such as bioadhesive polymers [182,183]. These polymers represent a crucial class of excipients used in the formulation of numerous drug delivery systems, including solid dosage forms such as tablets, capsules, powders, multi-compartment systems, thin strips, inserts, and liquid dosage forms such as solutions, suspensions, gels, and foams. Therefore, bioadhesive excipients such as polymers could be utilized for multiple drug delivery systems and could be used to target multiple positions. Bioadhesive polymers could be categorized into two main categories: natural polymers and synthetic polymers (Figure 7) [182,183,184].

Natural bioadhesive polymers include chitosan, alginate, pectin, xanthan gum, tragacanth, and silk polymers (Table 6) [185]. Chitosan, the most common one, as a natural polysaccharide derived from shellfish, is known for its higher biocompatibility and biodegradability. It has been widely utilized for multiple pharmaceutical and medical applications such as wound closure and hemostasis and exhibits higher solubility in aqueous solutions with a pH below 6.5 [186,187]. Lehr et al. investigated the in vitro mucoadhesive properties of chitosan by evaluating the detachment forces of swollen chitosan polymer films from pig intestinal mucosa in saline media [188]. In contrast to natural polymers such as hydroxypropyl cellulose and carboxymethyl cellulose, which demonstrated minimal mucoadhesion, the positively charged polymer chitosan exhibited significantly greater mucoadhesion compared to polycarbophil [188]. Further studies by He et al. measured the mucin absorbed on chitosan microspheres using turbidimetric methods, revealing strong interactions between mucin and chitosan microspheres with varying degrees of crosslinking [189]. Chitosan microspheres also demonstrated retention in biological tissues. Additionally, Sogias et al. explored the interactions between gastric mucin and chitosan, finding that a reduction in the number of amino groups enhanced the pH solubility range of chitosan while diminishing its capacity to aggregate mucin [187]. Chitosan as an excipient is widely utilized as a bioadhesive polymer in various dosage forms, such as tablets, inserts, films, and gels, for multiple drug delivery systems in the oral, buccal, nasal, vaginal, and ocular routes (Figure 7) [187].

Chitosan, an adhesive hydrogel, demonstrates strong mucoadhesive properties with minimal cytotoxicity [186]. Research indicates that incorporating catechol-containing compounds such as Dihydroxyphenylalanine (DOPA), dopamine, and hydrocaffeic acid (HCA) into chitosan enhances these properties [190]. The DOPA and dopamine mixtures with chitosan exhibit the highest hydrogel factor, while HCA–chitosan hydrogels, due to electrostatic interactions, show slow catechol release and increased mucoadhesion [190]. Additionally, HCA oxidation during mucosal contact further improves adhesion [190]. Inspired by mussel adhesion mechanisms, the chitosan–catechol conjugate, synthesized via various methods including chemical and enzymatic, exhibits remarkable solubility, tissue adhesion, and mechanical strength [191]. The chitosan–catechol conjugate can be fabricated into diverse forms like hydrogels and nanoparticles. The bio-inspired designs, mimicking natural adhesive systems such as those in mussels and insect cuticles, reveal enhanced viscosity, adhesive strength, and stability in physiological conditions, although chitosan’s load-bearing capabilities in hydrated conditions remain limited [191].

On the other hand, numerous mucoadhesive polymers, either natural or synthetic, are available for bioadhesion and mucoadhesion functions (Table 6). Mussel Adhesive Protein (MAP), a 130 kDa protein with mucoadhesive properties derived from the blue mussel Mytilus edulis, is facilitated in its interaction with mucosal surfaces by the presence of dihydroxyphenyl alanine (Dopa) [192]. MAPs contain a high amount of a unique catecholic amino acid, Dopa, in their protein sequences. Catechol provides strong and durable adhesion to different substrate surfaces and supports the curing of adhesive plaques. These properties have a great impact on the mucoadhesion properties. Alginate is a natural polymer that is both biodegradable and mucoadhesive. It is utilized in drug delivery systems because, when mixed with divalent ions like calcium, it may produce stiff, porous gels that increase its usefulness in a range of biomedical applications [193]. The synthetic polymer known as alginate–PEGAc, which combines alginate and PEG, exhibits efficient mucoadhesion by means of Michael-type addition reactions and PEG’s interpenetration with the mucosal layers. This polymer has an impact on the food and biomedical industries and is employed in controlled drug release. It is biocompatible, bioactive, and has minimal toxicity [194,195]. Inspired by transdermal patch designs, the Pectin–Sodium Carboxymethyl Cellulose System forms a robust mucoadhesive matrix that can tolerate large forces, allowing for oral drug delivery [196]. The mucoadhesive qualities of Carbopol 934P, a gel with high viscosity and flexibility, have been investigated, especially in relation to drug delivery systems. It has been demonstrated to have higher adhesion under restricted water settings and lower fasting blood glucose levels [197].

**Table 6:** Bioadhesive and mucoadhesive polymers and their applications.

Drug	Polymer ^1^	BCS Class	Formulation	Biofunctional Application	Route	Ref.
Doxazocin	Chitosan	BCS III	Hydrogel	Bioadhesive polymeric-based drug delivery systems enable sustained, prolonged, and targeted in vivo drug release through mucoadhesion, diffusion, swelling, and erosion mechanisms. Their mucus-binding properties facilitate controlled drug release across gastric and intestinal environments, enhancing drug permeation, bioactivity, and local persistence while ensuring gradual release from polymeric networks for improved therapeutic efficacy.	Implant	[198]
Doxorubicin		BCS IV	Hydrogel		In vitro	[199]
Gentamicin		BCS III	Hydrogel		Ear	[200]
L-asparaginase		--	Nanoparticles		In vitro	[201]
VEGF^1^	Alginate	--	Hydrogel		Implant	[202]
Curcumin		BCS IV	Nanoparticles		In vitro	[203]
Doxorubicin		BCS IV	Nanoparticles		In vitro	[204]
Tilmicosin		BCS III	Nanogel		Buccal	[205]
Vancomycin		BCS III	Microparticles		In vitro	[205]
Curcumin	Pectin	BCS IV	Hydrogel beads		Oral	[206]
Vancomycin		BCS III	Hydrogel/scaffold		Oral	[207]
Ciprofloxacin	Alg-PEG	BCS III	Polymeric system		In vitro	[208]
Cisplatin	Gelatin	BCS III	Microparticles		Injection	[209]

^1^ VEGF denotes Vascular Endothelial Growth Factor and Alg-PEG denotes alginate–PEGAc polymer.

### 4.2. Biofunctional Amino Acids, Surfactants, and Cyclodextrins

Oral drug delivery is widely favored due to its precise dosing, cost efficiency, and enhanced patient adherence [210]. For effective absorption, the drug must dissolve in GIT fluids and permeate biological membranes, making membrane permeability a key factor for oral drugs and other drugs that act after crossing biological membranes such as nasal, topical, and rectal routes, as outlined in the BCS [9,211]. Recent advancements have focused on the use of AAs to improve drug permeability [212,213]. AA-based prodrugs, such as floxuridine-L-ILE, have demonstrated significantly increased intestinal absorption and bioavailability due to oligopeptide transporters [214]. Similarly, salt formation techniques using anionic AAs like ASP and GLU have enhanced the permeability and bioavailability of numerous drugs [215]. Studies have also explored the ion-pairing of AAs with drugs like insulin to improve lipophilicity and transcellular transport, particularly with AAs like LYS, HIS, ASP, and GLU, which enhance permeability through ion-pairing mechanisms [108] (Table 3). AAs have a great impact on the solubility, absorption, and permeability of the poorly soluble non-ionizable drug carbamazepine compared to cyclodextrin complexation [103].

Finally, several studies highlight the effectiveness of AAs in enhancing therapeutic outcomes across various drug formulations. A notable example is the naproxen-L-ALA cocrystal, which, in a 1:1 ratio-based tablet formulation, demonstrated improved pharmacokinetic profiles and enhanced oral bioavailability compared to pure naproxen. This cocrystal formulation not only increased plasma drug concentration, prolonging therapeutic activity, but also reduced gastric irritation due to a faster gastric emptying rate [216]. Similarly, cocrystals of itraconazole with AAs such as ASP, GLY, PRO, and SER were found to enhance anti-fungal efficacy against *Aspergillus niger* and *Candida albicans*, achieving greater inhibition at lower concentrations compared to the drug alone [217]. Additionally, the indomethacin-L-PRO zwitterionic cocrystal showed improved solubility and permeability, leading to faster absorption, a quicker pharmacological response, and prolonged action due to an extended half-life [217]. AAs have also been employed as selectively targeted delivery systems. For example, the ASP–doxorubicin system exhibited a longer circulation time, increased tumor accumulation, and enhanced selective cytotoxicity against human hepatoma cells by targeting L-type AA transporter 1, which is overexpressed in tumor cells [218]. These findings underscore the potential of AAs as biofunctional excipients in optimizing drug delivery and therapeutic efficacy (Table 3).

From the point of CDs, nasal drug delivery is a highly promising method due to the nasal cavity’s extensive surface area and rich vascularization, which enhances drug absorption. The nasal route bypasses first-pass metabolism, leading to lower drug dosages, rapid therapeutic levels, and quick pharmacological effects [219]. Given β-CD’s low water solubility, various derivatives have been developed to improve solubility. In nasal delivery, derivatives such as dimethyl-β-CD, randomly methylated β-CD, and hydroxypropyl-β-CD have been utilized [220]. Cyclodextrins serve as absorption enhancers, facilitating the delivery of numerous compounds, including hormones, peptides, and proteins [221]. For instance, α-CD enhances the nasal absorption of leuprolide, while dimethyl-β-CD improves the bioavailability of insulin when administered intranasally [135]. The nasal delivery of morphine has shown superior systemic availability compared to oral and rectal routes with enhanced analgesic effects [135]. Additionally, CDs like heptakis (2,6-di-O-methyl)-β-CD improve the nasal absorption of opioids, while others may reduce permeability and plasma concentrations [222] (Table 4).

In the same way, in ophthalmology, drugs are administered either locally or systemically to the eye, with local applications often resulting in very low ocular bioavailability (<5%). The cornea, conjunctiva, and sclera act as major barriers to drug permeability, alongside the hydrophilic mucin film and aqueous tear layer [135]. Effective ophthalmic drugs must balance hydrophilic properties to permeate the eye’s aqueous environment and hydrophobic characteristics to penetrate ocular barriers. A key requirement for ophthalmic formulations is non-irritation, as irritation can trigger blinking and tearing, leading to drug washout. Cyclodextrins (CDs) and their chemical derivatives are utilized in ophthalmic preparations to enhance drug solubility, stability, and reduce irritation. For example, 2-hydroxypropyl-β-CD and sulfobutyl-β-CD are well tolerated in eye drops, improving the ocular bioavailability of hydrophobic drugs by maintaining them in solution at the corneal surface [223]. Combining CDs with polymers like HPMC further enhances the surface delivery of drugs like carbonic anhydrase inhibitors [224] (Table 4).

Additionally, over the past decade, dermal or topical drug delivery has earned a significant attention due to its success in delivering a wide range of drugs. However, the stratum corneum, the outermost layer of the epidermis, acts as a major barrier, hindering drug transport through the skin [135]. To overcome this, various strategies have been developed to improve drug absorption, target tissues, and improve drug retention within the skin [135]. Cyclodextrins (CDs) and their derivatives play a crucial role in enhancing dermal drug delivery by improving solubility, stability, and transdermal absorption; sustaining drug release; and minimizing side effects [225]. The choice of vehicle is critical; for example, hydrophilic CDs paired with water-based ointments can enhance corticosteroid release, whereas other bases like propylene glycol may delay it [226]. CDs also interact with skin components, potentially altering skin barriers to facilitate absorption and reduce irritation [227]. For instance, methylated β-CD disrupts skin lipid barriers, enhancing drug absorption. CDs are also employed in liposomal drug delivery systems, improving solubility, stability, and controlled release, as seen with ketoprofen and Prostaglandin E1 complexes [228] (Table 4).

Finally, surfactants can act as penetration enhancers in topical drug delivery systems. The main mechanisms through which surfactants interact with skin are through their actions on lipids, proteins, and living cells. Surfactant molecules initially diffuse through the lipid layers to reach protein-rich regions inside the stratum corneum. This causes the disruption of lipid organization and a decrease in corneocyte adhesion, which in turn causes protein denaturation, stratum corneum swelling, and greater protein accessibility [229]. In this instance, anionic surfactants interact with skin keratin and solubilize less soluble proteins, hence enhancing the denaturation of proteins and changing the activity of enzymes [230]. Furthermore, surfactants incorporate into lipid bilayers, disrupting the lipid barrier of the skin and influencing its permeability. Higher concentrations of surfactants can trap permeants within micelles, decreasing permeability, whereas lower quantities may emulsify stratum corneum lipids and promote permeability [231]. It has been demonstrated that sodium lauryl sulfate (SLS) increases lipid fluidity, improving skin penetration and permitting diffusion through several channels [232]. After the lipid barrier is breached, surfactant monomers can interact with keratinocytes and Langerhans cells, two types of live cells found in the epidermis, possibly resulting in biological alterations without thickening the epidermis [230].

## 5. Biofunctional Excipients Added to Enhance Drug Release and Kinetic Profiles

Modified-release drug delivery systems aim to maintain constant plasma drug levels for prolonged therapeutic effects. However, some drugs require excipients that respond to specific physiological conditions to regulate drug release based on patient needs. In such cases, programmability is crucial for restoring biological homeostasis. The conventional therapeutic window should account for the timing of drug delivery. Smart polymers enable responsive drug release, such as insulin delivery triggered by blood glucose fluctuations. Recent advances in biocompatible polymers have led to systems where drug release is controlled by environmental stimuli like temperature, pH, enzymes, or external triggers such as ultrasound, magnetic fields, or electric stimulation. These stimuli-sensitive polymers include interpolymeric complexes, graft copolymers, and hydrogels. Their ability to respond to specific conditions enhances drug delivery precision, improving therapeutic outcomes. Various polymers with tailored sensitivity have been previously reviewed, highlighting their potential in innovative pharmaceutical applications [233].

### Smart or Intelligent Polymers

Recently, a novel category of polymers has emerged, noted for their exceptional and remarkable responsive behavior. Stimuli-responsive polymers provide a promising drug delivery system capable of administering therapeutics at a controlled rate while maintaining stability and biological activity. These polymers undergo significant and profound physicochemical transformations and changes in response to alterations in their local environment [234]. This “responsive behavior” is characterized by their rapid and reversible nature, allowing the polymers to revert to their original states once the external stimulus is removed. Hence, these materials are termed “smart”, “intelligent”, or “stimuli-sensitive” due to their capability to detect and respond to minor environmental changes [234,235]. Various stimuli, including physical (temperature, light, ultrasound, electricity, magnetic fields), chemical (pH and organic phases), and biological (biomolecules), can act as triggers to elicit a desired response in these polymers. These stimuli can be either naturally occurring within the body’s internal physiological environment or introduced externally through devices designed to generate these stimuli. These polymers act as biofunctional polymers, as they could modify or alter drug release and pharmacokinetic profiles. Also, they could modify drug absorption and permeation rates (Figure 7) [234,235].

The uniqueness of these polymers is characterized by their nonlinear response to minimal stimuli, as described previously, leading to substantial macroscopic alterations in their structure. The primary advantages of drug delivery systems utilizing smart polymers include a decreased dosing frequency, simplified preparation, extended release of the incorporated drug, maintenance of the desired therapeutic concentration with a single dose, minimized adverse effects, and enhanced drug stability [236,237]. For a smart polymer to be effective, it must exhibit several essential characteristics: it should be inert, biodegradable, and biocompatible; possess a controlled release profile; and have a high drug loading capacity. Moreover, it must be free from adverse properties such as systemic toxicity, carcinogenicity, immunogenicity, and reproductive toxicity. Additionally, the polymer should demonstrate excellent stability to ensure reliable performance in drug delivery applications [238].

Thermo-sensitive or temperature-sensitive polymers exhibit significant and considerable solubility changes in response to minor temperature variations, with aqueous solutions undergoing temperature-dependent and reversible sol–gel transitions near body temperature. This process, which helps maintain the physicochemical stability and biological activity of the incorporated drug, is primarily influenced by the hydrophilic to lipophilic ratio on the polymer chain and the free energy of mixing [239,240]. Thermo-sensitive polymers commonly feature hydrophobic groups such as methyl, ethyl, and propyl groups, and are characterized by critical parameters including the lower critical solution temperature (LCST) and the upper critical solution temperature (UCST). LCST polymers become hydrophobic and insoluble above their LCST, leading to phase separation, whereas they remain soluble below this temperature. This behavior is due to the entropy-driven nature of the system, where the free energy decreases as temperature increases, resulting in higher entropy relative to enthalpy. LCST systems, preferred in drug delivery technologies, facilitate polymeric micelle packing and coil-to-helix transitions at critical temperatures (Figure 7) [240,241].

A study by Rahimi et al. (2008) investigated how Poly(N-isopropylacrylamide-co-acrylamide-co-allylamine) nanoparticles were synthesized by free radical polymerization containing doxorubicin [242]. Nanoparticles showed low cytotoxicity and were conjugated effectively with IgG-Texas Red. Doxorubicin release increased at 41 °C, demonstrating temperature sensitivity and the potential for drug delivery applications [242].

Prominent and most common examples of LCST polymers include Poly(N-isopropylacrylamide) (PNIPAM), which exhibits a sharp LCST at 32 °C, adjustable to body temperature with additives [240,243]. Despite its effectiveness in drug delivery, PNIPAM’s application is limited by cytotoxicity and non-biodegradability. Advances in reducing initial burst drug release have been achieved through optimizing the hydrophilic and hydrophobic segment ratios, as demonstrated by triblock polymers like PCL-PEG-PCL, which provide sustained drug release [244]. Other examples of temperature polymers include poly(organophosphazene), cyclotriphosphazenes with poly(ethylene glycol) and amino acid esters, block copolymers of poly(ethylene glycol)/poly(lactic-co-glycolic acid), PEG, Poly(propylene glycol) (PPG), PMAA, PVA, various silk-elastin-like polymers, Poly(silamine), Poly(vinyl methyl ether) (PVME), Poly(vinyl methyl oxazolidone) (PVMO), PVP, Poly(N-vinyl isobutyl amid), Poly(N-vinylcaprolactam) (PVCL), Poly(siloxyethylene glycol), Poly(dimethylamino ethyl methacrylate), triblock copolymer poly(DL-lactide-co-glycolide-b-ethylene glycol-b-DL-lactide-co-glycolide) (PLGA-PEG-PLGA), cellulose derivatives, alginate, gellan, and xyloglucan (Table 7).

A thermo-gelling Poly(N-isopropylacrylamide)–chitosan (PNIPAAm-CS) hydrogel was developed to sustain the delivery of timolol maleate for reducing intraocular pressure. Compared to conventional eye drops, the hydrogel improved drug bioavailability (2-fold increase) and enhanced therapeutic efficacy [245]. Also, 5-FU was encapsulated into a PNIPAAm hydrogel, and drug release was measured using UV spectrophotometry. Faster release occurred at higher temperatures (at 37 °C), confirming that the hydrogel improved controlled drug delivery [246]. A thermo-gelling system was formed by copolymerizing 3-caprolactone, 1,4,8-trioxa [4.6] spiro-9-undecanone, and poly(ethylene glycol) hydrogel at 37 °C. It carried chemotherapeutic drug-loaded nanoparticles and was used to treat bladder tumors in mice, generating a 4.8-fold increase in reactive oxygen species compared to free drugs [247].

There are also various types of polymers that respond to multiple stimuli, including photo-sensitive polymers, electric-sensitive polymers such as sulfonated polystyrenes, and magnetic-sensitive polymers such as poly(ethylene-co-vinyl acetate) [235]. Magnetic-responsive polymer composites (MRPCs), created by combining polymers with magnetic nanoparticles like Fe₃O₄, offer fast response times and diverse applications. These include mechanical actuation, biomedical targeting, and controlled drug release [248]. These polymers can significantly influence drug release and pharmacokinetic profiles. Additionally, these polymers have broad applications in targeted drug delivery systems and biomedical imaging. Their multifunctional nature enables precise control over drug release, thereby enhancing therapeutic efficacy and minimizing side effects.

**Table 7:** Examples of biofunctional thermo-sensitive polymers for drug release modification.

Drug	BCS Class	Polymer ^1^	Biofunctional Applications	Formulation	Ref
Doxorubicin	BCS IV	PNIPAM	Modified and targeted in vivo drug release based on body temperature. PNIPAM polymer collapsed and modified the drug release at temperatures above 32 °C.	Nanoparticles	[243]
Doxorubicin	BCS IV			Nanotubes	[249]
Ibuprofen	BCS II			Hydrogel	[250]
5-Fluorouracil	BCS I			Hydrogel	[250]
Paclitaxel	BCS IV			Liposomes	[251]
GH	Biologic	PCL-PEG-PCL	Modified in vivo drug release controlled via the balance between the hydrophilic segment (PEG) and hydrophobic segment (PCL) with thermo-sensitive properties.	In situ gel	[252]
Silver	Metal			Micelles	[253]
Diclofenac	BCS II			Hydrogel	[254]
Doxorubicin	BCS IV	PLGA-PEG-PLGA	Targeted, controlled, and sustained in vivo drug delivery via the benefits from the hydrophobicity of PLGA and the biocompatibility of the PEG segment.	Hydrogel	[255]
Dexamethasone	BCS II			Hydrogel	[256]
Cyclosporine	BCS IV			Hydrogel	[257]
Naltrexone	BCS I			Hydrogel	[258]
Simvastatin	BCS II			Hydrogel	[259]
Ketorolac	BCS II			Hydrogel	[260]
Metronidazole	BCS I	PVME	Controlled in vivo drug release	Hydrogel	[261]
Doxorubicin	BCS IV	PNVCL	Controlled in vivo drug release	In situ gel	[262]
5-Fluorouracil	BCS I	PolyDMAEMA	Modified in vivo drug release	Nanocarriers	[263]
Curcumin	BCS IV	PVA	Controlled in vivo drug release	Nanofibrous	[264]
Doxorubicin	BCS IV	PNIAM-PCL	Sustained in vivo drug release	Porous film	[265]

^1^ PNIPAM: Poly(N-isopropylacrylamide); PCL-PEG-PCL: block copolymer of poly caprolactone–polyethylene glycol; PLGA-PEG-PLGA: block copolymers of poly(ethylene glycol)/poly(lactic-co-glycolic acid); PVME: Poly(vinyl methyl ether); PNVCL: Poly(N-vinylcaprolactam); polyDMAEMA: Poly(2-(dimethylamino)ethyl methacrylate); PVA: polyvinyl alcohol; and PNIAM-PCL: block copolymer of Poly(N-isopropylacrylamide) and poly caprolactone.

pH-sensitive polymers represent another category of smart or intelligent polymers that respond to pH changes within the physiological range of the GIT compartments [266]. All pH-sensitive polymers contain pendant acidic or basic groups that enable them to either accept or donate protons in response to variations in the surrounding pH. This interaction results in modifications to their structural and physical properties, including polymer chain conformation, surface activity, and solubility [266]. The specific nature of these changes is determined by the structure of the pH-responsive material (Figure 7). For example, variations in pH can induce phenomena such as chain collapse or extension, polymer flocculation, and precipitation. Conversely, pH-responsive block copolymers or cross-linked structures exhibit self-assembly behaviors, including the formation of unimers, micelles, vesicles, gels, and swelling or deswelling in response to pH fluctuations [267] (Table 8).

These polymers, which incorporate weak acidic or basic groups within their structure, ionize their functional groups in response to environmental pH fluctuations, modifying their polyelectrolyte structure. Polyanions or polyacids, containing ionizable acid groups like carboxylic acid (COOH), sulfonic acid (SO_3_H), or phosphonic acid (PO_3_H_2_), and polycations or polybases containing basic groups such as amines (NH_2_), respond to pH variations by altering their structural configuration. pH-responsive polymers include various examples such as pH-responsive dendrimers, i.e., poly-amidoamide (PAMAM), poly(propyleneimine) dendrimers (PEIs), poly(L-lysine) ester, poly(propyl acrylic acid), poly(ethacrylic acid), Carbopol^®^, polysilamine, Eudragit^®^ S-100, Eudragit^®^ L-100, chitosan, poly(methacrylic acid) (PMMA), PMAAPEG copolymer, maleic anhydride (MA), and N,N-dimethylaminoethyl methacrylate (DMAEMA) (Table 8).

These polymers offer diverse applications and biomedical uses, including targeted drug delivery, colon-specific systems, tumor-targeted therapies, controlled drug release, and gene delivery, owing to their responsiveness to pH variations within lysosomal compartments. A notable drawback of pH-responsive polymers is their dependence on environmental pH, which can vary significantly with the severity of disease or the proximity to diseased tissues. This variability can pose challenges in maintaining the structural integrity of the delivery system throughout the administration process. Additionally, pH-sensitive systems can be inadvertently activated during implantation or administration, increasing the risk of off-target delivery. Addressing these challenges, while also enhancing the existing advantages, presents opportunities for future research and development in pH-responsive polymers for drug delivery applications [268].

There are also various types of polymers that respond to multiple stimuli, including photo-sensitive polymers, electric-sensitive polymers such as sulfonated polystyrenes, magnetic-sensitive polymers such as poly(ethylene-co-vinyl acetate), and time-dependent polymers such as Eudragit RS100 and RL100 [235]. These polymers can significantly influence drug release and pharmacokinetic profiles. Additionally, these polymers have broad applications in targeted drug delivery systems and biomedical imaging. Their multifunctional nature enables precise control over drug release, thereby enhancing therapeutic efficacy and minimizing side effects [269,270].

**Table 8:** Examples of biofunctional pH-sensitive polymers for drug release modification.

Drug	BCS Class	Polymer ^1^	Biofunctional Applications	Formulation	Ref
Psoraldin	--	Eudragit S100	Enhanced in vivo drug bioavailability, drug release profiles, and drug targeting properties. Eudragit S100 acts as a protective polymer matrix for the drug at acidic pHs, such as in the stomach, and gradually releases the drug at neutral and slightly alkaline pHs, such as in the small intestine and the end of the ileum. These biofunctions allow Eudragit S100 to control drug release, deliver the drug to the colon, protect the drug from the harsh environment of the stomach, and finally enhance drug bioavailability.	Nanocapsules	[271]
Zaleplon	BCS II			Microspheres	[272]
5-Fluorouracil	BCS I			3D-Printed Tablets	[273]
Prednisolone	BCS I			Microsponges	[274]
Budesonide	BCS II			Nanocapsules	[275]
Etoricoxib	BCS II			Nanoparticles	[276]
Insulin	Biologic			Nanoparticles	[277]
Sulfasalazine	BCS IV			Bilayer tablets	[278]
Flurbiprofen	BCS II			Coated matrix tablet	[279]
Mesalamine	BCS IV			Tablets	[280]
Azithromycin	BCS III	Eudragit L100	Enhanced delayed, controlled, and ocular drug delivery via drug protection at lower acidic pHs such as in the stomach and release of the drug at higher acidic pHs such as in the upper small intestine. Also, provides precise temporal drug release for ocular drug delivery (ocular pH like pH of Eudragit L100 degradation).	Polymeric Inserts	[281]
Dexamethasone	BCS II			Nanoparticles	[282]
Enoxaparin	Biologic			Nanoparticles	[283]
Fenofibrate	BCS II			Nanoparticles	[284]
Rosuvastatin	BCS III			Nanoparticles	[285]
Duloxetine. HCL	BCS II			Tablets	[286]
Quercetin	BCS IV			Nanoparticles	[287]
Senna	--			Nanoparticles	[288]
Doxorubicin	BCS IV	PAMAM	Cancer treatment.	Dendrimers	[289]
Diclofenac	BCS II	Carbopol	Controlled in vivo drug release.	Hydrogel	[290]
Sulfacetamide	BCS III		Ocular drug delivery.	In situ gel	[291]
Naproxen	BCS I		Ocular drug delivery.	In situ gel	[292]
Doxorubicin	BCS IV	DMAEMA	Regulation of in vivo drug release.	Hydrogel	[293]

^1^ PAMAM: Polyamidoamine dendrimers; DMAEMA: 2-(dimethylamino)ethyl methacrylate; Eudragit is a polymethacrylate copolymer.

## 6. Favorable and Unfavorable Interactions of Biofunctional Excipients

As discussed previously, functional or biofunctional excipients can interact favorably with active pharmaceutical ingredients (APIs) or biological systems in multiple ways. They may enhance drug solubility, permeability, penetration, release, and bioavailability. Additionally, biofunctional excipients can improve drug bioavailability by protecting against degradation, inhibiting metabolic pathways, and modulating pH. Unfortunately, biofunctional excipients can interact unfavorably with APIs or biological systems, leading to reduced drug bioavailability.

### 6.1. Biofunctional Excipients and Drug Metabolism

One of the most significant concerns regarding excipients is their potential to influence active ingredients and function as biofunctional substances by affecting drug metabolism through either inhibition or induction. There are multiple excipients that have been investigated for their impact upon Cytochrome P450 (CYP450), such as surfactants, lipids, polymers, and others. Substances that influence CYP450 activity can alter drug metabolism, affecting bioavailability. For instance, propylene glycol in liquid acetaminophen inhibits CYP2E1, reducing toxicity compared to solid formulations [294].

Surfactants, commonly used for solubilization, also affect CYP activity. These include anionic, cationic, zwitterionic, and non-ionic surfactants [295]. Cremophor EL, a non-ionic surfactant, solubilizes hydrophobic drugs but inhibits CYP3A4 and CYP2C9 in a concentration-dependent manner. A study by Mudra et al. (2010) reported the 20.7% and 21.8% inhibition of CYP3A4 in situ and in vitro, respectively, by Cremophor EL at 47.5 μg/mL [296]. Additionally, Cremophor EL also inhibits 7-ethoxycoumarin metabolism, reducing CYP1A2, 1A1, and 2B activities as its concentration increases [297,298]. Gonzalez et al. found that at concentrations above its critical micellar concentration (CMC), Cremophor EL significantly reduced midazolam (MDZ) metabolism in rat hepatocytes and microsomes, lowering intrinsic clearance by up to 54.9% [299]. Tween 80, a widely used non-ionic surfactant, inhibits CYP3A4 and CYP2C9 with IC50 values of 0.40 mM and 0.04 mM, respectively [296]. In a study, an increasing Tween 80 concentration in SMEDDS reduced 1′-OH-MDZ production from 80% to 30% [297]. In rat hepatocytes, Tween 80 significantly reduced MDZ clearance at concentrations above its CMC [300]. Tween 20, a related surfactant, was a more potent CYP inhibitor, reducing 7-ethoxycoumarin metabolism from 66% to 56% at concentrations between 0.03% and 0.10% [298]. Triton X-100 (TX-100), another non-ionic surfactant, strongly inhibits CYP enzymes, reducing 7-ethoxycoumarin metabolism by 54% and midazolam by 99.8% [298,301]. At 0.05 mM, TX-100 completely inhibited CYP activity in Prochilodus scrofa [302].

Polymers used in pharmaceuticals also affect CYP activity. Methoxy poly (ethylene glycol)-poly(ε-caprolactone) (mPEGx-PCLx) copolymers showed concentration-dependent CYP inhibition, with mPEG2k-PCL2k causing the most significant reduction [303]. Martin et al. found that nine out of ten tested polymers inhibited various CYP isoforms, with polyethylene glycol (PEG) downregulating CYP2E1, 3A5, 2C9, 2C19, and 2D6, and Pluronic F68 inhibiting CYP2E1, 3A4, and 3A5 [304]. Other polymers such as polyvinyl acetate (PVA), hydroxypropyl methylcellulose (HPMC), and polyvinyl pyrrolidone (PVP) also showed inhibitory effects [235]. Excipients significantly impact drug metabolism by inhibiting CYP enzymes. Their effects depend on concentration, surfactant type, polymer structure, and experimental conditions [305].

### 6.2. Biofunctional Excipients and Intestinal Transporters

Excipients also modulate intestinal carrier-mediated transport. Influx transporter interactions may decrease permeability, while efflux transporter interactions may increase it [306]. P-glycoprotein (P-gp), an efflux transporter expressed on enterocytes, interacts with multiple excipients. Polysorbate 80 significantly enhances digoxin bioavailability by inhibiting P-gp, and oral AUC increases by 30% with 1% Tween 80 and by 61% with 10% Tween 80 [307]. Surfactants with a high hydrophilic–lipophilic balance, including Span 80, Brij 30, and sodium lauryl sulfate, effectively inhibit P-gp, increasing drug bioavailability [308]. Polyethylene glycol (PEG) derivatives and D-Tocopherol Polyethylene Glycol 1000 Succinate also reduce P-gp activity [309]. Pluronic block copolymers inhibit P-gp and multidrug resistance proteins while reducing intracellular ATP levels [310]. Surfactants such as Polysorbate 20 and Cremophor EL enhance drug permeability by inhibiting ATP-binding cassette transporters [311].

Cyclodextrins also interact with secretion transporters, particularly P-gp and MRP2, increasing tacrolimus and vinblastine bioavailability via enhancing hydrophobic drug solubilities and inhibiting post-translational P-gp and MRP2 transporters as it reduces cholesterol content in caveolar membranes (P-gp localized in cholesterol-rich caveolae) [312]. Some surfactants indirectly inhibit transporters by modifying membrane fluidity and altering the cellular microenvironment [313]. Excipient-induced permeability modifications pose risks for narrow therapeutic index drugs. Increased permeability can lead to toxicity, while decreased permeability reduces efficacy. Digoxin, a P-gp substrate, exemplifies this issue, as Cremophor RH40 increases its bioavailability by 20% [314]. Permeability enhancers raise concerns regarding irreversible epithelial damage and the unintended co-absorption of harmful substances [315]. Sodium caprate and melittin increase polar sugar permeability but cause mucosal damage [316]. Permeability enhancers vary in toxicity, with cyclodextrins exhibiting higher toxicity than bile salts and medium-chain fatty acids [317]. Some lactose-based non-ionic surfactants induce cytotoxicity at high concentrations but are reversible permeability enhancers at lower levels [318].

### 6.3. Biofunctional Excipients and pH Modulation

The pH of the boundary layer, or stagnant diffusion layer, surrounding solid drug particles is critical for the dissolution rates of both weak acidic and basic drugs [319]. Including pH-modifiers in a formulation can enhance the dissolution rate of these drugs by modifying the pH of this layer or microenvironment. These excipients are blended with other ingredients before the formulation is compressed into tablets or encapsulated. Upon disintegration in the gastrointestinal tract, they release H+ or OH− ions, adjusting the pH of the stagnant diffusion layer to promote the dissolution of the drug by favoring its ionized form [320,321]. Weakly basic medications can dissolve more quickly when pH-adjusting excipients, such as organic acids like tartaric acid, citric acid, and carbonic acid, are added. These organic acids dissolve in the gastric juices following the disintegration of a solid dosage form, lowering the pH of the stationary diffusion layer that surrounds each drug particle. This makes the milieu ideal for basic medication solubility and subsequent absorption. On the other hand, because they mostly exist in their ionized form, acidic medicines acquire superior solubility and dissolution rates in an alkaline microenvironment [320,321].

A study by Adachi et al. stated that the incorporation of organic acids might enable effective treatment outcomes regardless of the patients’ stomach pH. Furthermore, the oral delivery of ketoconazole (KZ)/citric acid (CA) granules at gastric acidic pH circumstances resulted in the increased bioavailability of KZ compared to KZ alone, with the AUC rising from 4185 to 8697 µg·min/mL. As a result, after oral administration, CA increased KZ’s rate of absorption and disintegration [322]. A study by Taniguchi et al., stated that the addition of p-toluenesulfonic acid to the dipyridamole formulation as a microenvironmental pH-modifier should help increase the drug’s therapeutic potential for treating hypochlorhydric patients. In hypochlorhydric rats, oral dipyridamole (10 mg DP/kg) showed a 40% reduction in AUC₀_–_₃ compared to normal rats. In contrast, dipyridamole/p-toluenesulfonic acid maintained AUC_0–3_ levels similar to those in normal rats [323]. Another study by Halder et al., observed that the inclusion of citric acid (CA) as a pH-modifier in carvedilol amorphous solid dispersions (CAR-ASD) could ensure the consistent pharmacokinetic behavior of carvedilol, even in conditions of reduced stomach acidity (hypochlorhydria) [324]. Also, Tran et al. evaluated six alkalizers as pH-modifiers in the development of an SD formulation for telmisartan, a zwitterionic compound with pKa values of 3.5, 4.1, and 6.0. Telmisartan’s solubility is highly pH-dependent, with optimal solubility at both extreme pH levels but poor solubility within the pH range of 3 to 9. Their study identified magnesium oxide as the most effective alkalizer for this SD formulation, as it enhanced drug dissolution, optimized the microenvironmental pH, and maintained drug crystallinity (Table 9) [325,326].

A study demonstrated that incorporating sodium bicarbonate (NaHCO_3_) into paracetamol tablets significantly accelerated absorption, indicated by a lower T_max_ and higher C_max_, likely due to an enhanced in vivo dissolution rate, though this effect was not observed with calcium carbonate (CaCO_3_). Sodium bicarbonate may enhance paracetamol absorption by increasing the dissolution rate and raising stomach pH, leading to a higher proportion of a non-ionized drug that can better permeate gastric and intestinal tissues. With a pH around 8.3 and with paracetamol’s pKa at 9.5, about 6% of the drug becomes ionized, supporting faster absorption [327]. In another study, combining Na_2_CO_3_ and poloxamer 407 with aceclofenac improved its in vitro dissolution rate through pH modulation, altered drug crystallinity, and created a favorable nanoemulsion environment [328]. Additionally, Na_2_CO_3_ in aceclofenac bilayered tablets enhanced solubility, increased plasma concentration, and reduced gastrointestinal bleeding in dogs, likely due to pH effects and smaller drug particles (Table 9) [329].

**Table 9:** List of pH-modifiers and their applications as biofunctional excipients.

Drug	BCS Class	pH-Modifier ^1^	Formulation ^1^	Route	Biofunctional Applications	Ref.
Ketoconazole	BCS II	Citric acid	Granules	Oral	Enhanced oral bioavailability	[322]
Ketoconazole	BCS II	Tartaric acid	Granules	Oral	Enhanced oral bioavailability	[322]
Paracetamol	BCS II	NaHCO_3_	Tablets	Oral	Enhanced absorption rates	[327]
Aceclofenac	BCS II	Na_2_CO_3_	Tablets	Oral	Enhanced drug release	[329]
Dipyridamole	BCS II	p-toluenesulfonic acid	Granules	Oral	Enhanced dissolution rate	[323]
Carvedilol	BCS II	Citric acid	SD	Oral	Consistent pharmacokinetics	[324]
Telmisartan	BCS II	Magnesium oxide	SD	In vitro	Enhanced dissolution rate	[326]
Paracetamol	BCS II	CaCO_3_	Tablets	Oral	Enhanced drug absorption	[327]
Aceclofenac	BCS II	Na_2_CO_3_	Tablets	Oral	Enhanced drug release	[328]
Diltiazem	BCS I	Citric acid	Tablets	In vitro	Enhanced drug dissolution	[330]
Vinpocetine	BCS II	Citric acid	Tablets	In vitro	Enhanced drug dissolution	[331]

^1^ SD, solid dispersion; NaHCO_3_, sodium bicarbonate; Na_2_CO_3_, disodium carbonate; and CaCO_3_, calcium carbonate.

## 7. Regulatory Aspects of Excipients and Biofunctional Excipients

The Food and Drug Administration (FDA) evaluates excipients within the framework of drug product applications, including investigational new drug applications (INDs), new drug applications (NDAs), and biological license applications (BLAs). There is no independent regulatory process for excipients. Consequently, their development and approval are inherently linked to new drug formulations. Briefly, INDs allow the early-stage testing of new drugs, NDAs seek full market approval, ANDAs pertain to generic drugs, and BLAs cover biologic products, with excipients evaluated in each according to the product type and use. This regulatory model, where excipients are reviewed only as part of drug applications, forms the basis for similar practices in the European Union and Japan, ensuring consistency across major regulatory frameworks. Excipients do not receive standalone approval but are recognized when included in FDA-approved drug products. Those present in NDA- or ANDA-approved drugs are listed in the FDA’s Inactive Ingredient Database (IID). Excipients in vaccines licensed through the BLA pathway appear in Appendix B of the CDC’s Pink Book. Most small-molecule excipients in the Pink Book are also found in the IID, with exceptions such as D-mannose and L-asparagine. Radiopharmaceutical excipients and certain biologics may be absent from both databases. The FDA distinguishes between novel and new excipients. A novel excipient has no prior use in FDA-approved drugs or food. A new excipient lacks sufficient safety data for its proposed use but may already exist in approved drugs. For example, xylitol, an approved oral excipient, would be considered new if introduced in a parenteral formulation. The FDA’s definition of new excipients is broader than that of novel excipients [332].

Safety pharmacology studies must adhere to the International Council for Harmonization (ICH) guideline S7A. For excipients intended for short-term use, at least one of the following evaluations is required: (I) acute toxicology studies in both rodent and mammalian nonrodent species via the clinically relevant route (Center for Drug Evaluation and Research (CDER) Guidance for Industry on Single-Dose Acute Toxicity Testing); (II) absorption, distribution, metabolism, and excretion (ADME) studies in the species used for nonclinical safety evaluations (ICH S3A and S3B); (III) genetic toxicology assessments following ICH S2B; (IV) one-month repeat-dose toxicology studies in rodent and nonrodent species incorporating clinical pathology, histopathology, and toxicokinetic analyses; and (V) reproductive toxicology assessments per ICH S5A and S5B. For excipients intended for intermediate use, three-month repeat-dose toxicology studies are required in addition to the assessments, with further investigations necessitated in specific cases, such as for parenteral administration. Long-term-use excipients require all evaluations for short- and intermediate-term use, supplemented by a six-month repeat-dose toxicology study in rodents, chronic toxicology studies in nonrodent mammalian species, and carcinogenicity evaluations per ICH S1A [333,334]. On the other hand, these regulation guidelines face multiple challenges such as costs, formulation aspects, and the complexity of preclinical studies.

For excipients incorporated into injectable, topical (dermal, intranasal, intraoral, ophthalmic, rectal, or vaginal), or pulmonary drug products, safety assessments must encompass the following: (I) all applicable aforementioned studies; (II) sensitization assessments (e.g., guinea pig maximization test or murine local lymph node assay); (III) injectable excipients may require in vitro hemolysis studies, plasma creatine kinase analysis for intramuscular or subcutaneous administration, and protein-binding assessments; (IV) for excipients with systemic exposure via topical administration, toxicology studies via the intended and alternate administration routes may be necessary; and (V) ocular irritation studies for dermal and ophthalmic products [334]. For example, a study by El-Emam et al. on mizolastine-loaded solid lipid nanoparticles incorporated into ocular hydrogels utilized the Draize test to assess ocular irritation. The formulation was administered to rabbits, and observations indicated minimal irritation, supporting its potential safety for ophthalmic use [335].

In Europe, the European Medicines Agency (EMA) mandates excipient justification within the pharmaceutical development section of regulatory submissions. The rationale for excipient selection, concentration, and potential effects on drug performance and manufacturability must be addressed. Unlike the FDA, which evaluates excipients within the framework of drug product applications such as INDs, NDAs, and BLAs without an independent regulatory process for excipients, the EMA requires detailed justification for excipient use in submissions. Compatibility studies between the excipient and APIs and other excipients are required, alongside data on residual solvents in compliance with the CPMP/ICH/283/95 guideline. For excipients listed in the European Pharmacopoeia or national pharmacopeias, justification of specifications is generally unnecessary. Novel excipients require comprehensive characterization and safety data per the drug substance format guidelines. Considerations include bibliographic toxicology data, international specifications (e.g., FAO/WHO/JECFA), toxicological data specific to the dosage form and administration route, and regulatory documentation on chemical properties, stability, and impurity control per CPMP/QWP/130/96 [334,336].

## 8. Concluding Remarks and Future Perspectives

Biofunctional excipients are specialized additives used in pharmaceutical formulations to enhance the performance and effectiveness of active pharmaceutical ingredients (APIs). Unlike traditional excipients, which are typically inert and used to aid in the manufacturing process or to improve the taste and texture of medicines, biofunctional excipients have active roles in modifying drug properties.

An increasing recognition has emerged regarding the multifaceted in vivo effects exerted by pharmaceutical additives previously regarded as biologically inert excipients. This awareness has led to the emergence of biofunctional excipients to focus more on the drug’s biological behavior rather than on dosage form processing and manufacturability. These newly emerged biofunctional excipients encompass smart polymers, lipids, and other safe, natural additives capable of modulating drug solubility, release kinetics, permeability, and pharmacokinetic behaviors. This review provided a detailed discussion of the biofunctional applications of a wide range of excipients, including polymers, smart polymers, bioadhesive excipients, lipids, cyclodextrins, surfactants, and pH-modifiers. These biofunctional excipients provided self-assembled polymeric, lipid, and surfactant nanosystems that could offer innovative ways to solve biopharmaceutics’ inherent problems such as poor permeability and solubility.

In the future, further research is needed into solving the regulatory aspects and biofates of these biofunctional excipients, particularly those that are biocompatible, biodegradable, and can be tailored to specific drugs or delivery systems. The exploration of smart polymers and advanced drug delivery systems that respond to environmental triggers is a promising direction. Additionally, integrating biofunctional excipients with nanotechnology or personalized medicine approaches could be an area ripe for innovation. This review also calls for a deeper understanding of the mechanisms by which these excipients interact with drugs at the molecular level to optimize their effectiveness and safety in clinical applications. Furthermore, in-depth research should be conducted to detect and determine other unwanted biofunctions, such as metabolism, elimination, and distribution alterations. Currently, there are a broad range of excipient interactions with enzymes, proteins, carriers, cells, and other biochemical materials that might adversely affect drug functions or induce unwanted and unexplained adverse reactions.

## Figures and Tables

**Table 3:** Application of different amino acid biofunctional excipients in drug delivery systems.

Drug	AA Excipients ^1,2^	BCS Class	Formulation	Biofunctional Applications	Ref.
Curcumin	TYR, TRP, ARG	BCS IV	Binary Mixture	AAs show enhanced in vivo drug solubilization, permeation, stabilization, and bioavailability. AAs play a vital role in improving drug solubility, permeation, and stability, addressing key challenges in drug formulation, such as poor absorption and low bioavailability. AAs act as hydrophilic carriers and form ion pairs, enhancing the solubility and permeability of small molecules through AA carriers. They also improve drug stability by forming stable cocrystals and co-amorphous systems that prevent the recrystallization of poorly soluble drugs. Moreover, AAs facilitate drug transport across biological membranes, enhancing permeability and bioavailability. These properties make AAs effective solubility, permeation, and stability enhancers, crucial for optimizing drug formulations and therapeutic outcomes.	[109]
Carbamazepine	PHE, TRP	BCS II	Co-Amorphous		[110]
Naproxen	ARG	BCS II	Co-Amorphous		[104]
Ibuprofen	Cholinium	BCS II	Ionic liquids		[111]
Valsartan	HIS, ARG, LYS	BCS IV	Co-Amorphous		[112]
Genistein	ARG, GLU, LYS	BCS IV	Co-Amorphous		[113]
Telmisartan	ARG	BCS II	Co-Amorphous		[114]
Carvedilol	ASP, GLU	BCS II	Co-Amorphous		[115]
Glipizide	ARG, SER	BCS II	Co-Amorphous		[116]
Mebendazole	ARG, GLU	BCS IV	Co-Amorphous		[117]
Albendazole	GLU	BCS IV	Supramolecular		[118]
Griseofulvin	ASP, TRP, VAL	BCS II	Co-Amorphous		[119]
Insulin	ARG	BCS IV	Mixture		[91]
Meloxicam	SER, ARG	BCS II	Solid Dispersion		[120]
Curcumin	L-ARG	BCS IV	Salt Formation		[121]
Ketoprofen	L-LYS, L-ARG	BCS II	Salt Formation		[122]
Oxaprozin	ARG	BCS II	Ternary System		[123]

^1^ TYR: Tyramine, TRP: Tryptophan, ARG: arginine, PHE: Phenylalanine, HIS: Histidine, LYS: lysine, GLU: Glutamine, VAL: Valine, SER: serine, ASP: aspartic acid, Co-Amorphous: co-Amorphous formulations. ^2^ Seven amino acids were used in the following study, including glycine (GLY), alanine (ALA), serine (SER), arginine (ARG), cysteine (CYS), glutamic acid (GLU), and aspartic acid (ASP).

## Data Availability

Data will be available upon request.
